# High-Dimensional Linear Preference Model

**DOI:** 10.3390/e28091012

**Published:** 2026-09-11

**Authors:** Gil Ariel, Omer Peleg

**Affiliations:** Department of Mathematics, Bar-Ilan University, Ramat Gan 52900, Israel; omer.peleg@live.biu.ac.il

**Keywords:** multi-attribute utility, discrete choice, high-dimensional limit, preference markets, entropy, competitiveness

## Abstract

Multiple-criteria decision analysis (MCDA) is a branch of operations research concerned with choices based on several quantifiable factors. Here, we focus on high-dimensional markets, in which each available product is described by a large number of features. Taking a probabilistic approach, we define a market as a collection of alternatives in a decision-making scenario governed by a linear utility function. Analytic approximations for the market share and its moments are derived in the limit of a large population and a large number of measured features. We identify a single parameter, termed the degree of subjectivity, that places markets on a continuous spectrum ranging from fully objective to fully subjective. At an intermediate value, the market is competitive in the sense that it maximizes the entropy of the market-share distribution. Empirical analysis of several real markets indicates that they can indeed be classified by this parameter, yielding predictable decision patterns and a unified, relative measure of competitiveness across markets. Simulations involving non-linear utility functions and a trained machine-learning classifier provide preliminary evidence that similar behavior may also arise beyond the linear model, suggesting that the degree of subjectivity may be useful as a diagnostic in some broader multi-feature decision problems.

## 1. Introduction

Decision theory is an active topic of research due to its direct ties with consumer-based economies, the job market, politics, and art. Studying the statistics of preference could act as a window into the dynamics of modern society, where the selection of clothes, media, activities, and careers is larger than ever [1,2]. In finance, a well-known axis of preference is the trade-off between risk and expected return, as investors need to decide how much risk they are willing to accept [3]. Decision theory is also relevant to classification methods in data science. In classification problems, decision trees or other machine learning (ML) methods are used to assign an object into one out of a given finite set of categories. One can think of the classifier as “deciding” which category to choose based on a list of features that describe the object.

To describe the multi-attribute utility model [4,5], consider a group of individuals, each asked to pick one alternative (e.g., product) out of several possible ones. Each individual makes their choice according to their own preferences. This may lead to different rankings (preference orders) of the alternatives. A common assumption in decision theory is that individuals weight the properties of products, which are summarized to a numerical score, called the utility function. Each individual then picks the alternative that provides them with the highest utility. Our main interest is in finding the market share of each alternative—the rate at which it is chosen. Intuitively, the market share should depend on the characteristics of the alternative itself, on the characteristics of other available alternatives, and on the distribution of preferences in the population.

A thorough introduction and examination of the multi-attribute utility model can be found in references [4,5]. A methodology for the practical application of discrete choice modeling can be found in reference [6], and examples for real applications are found in references [7,8]. Although the literature on discrete choice and multi-attribute utility is extensive (see [9,10,11]), much of it is concerned with the utility functions themselves, fitting the data, or about the process of making a choice, while we are more interested in the market statistics.

Another central approach in the decision theory literature comes from random utility theory, which assumes some unobserved choice preference [12,13]. In particular, the multinomial logit model [14] can predict choice in cases with taste heterogeneity using likelihood estimation methods [12,15]. For decision problems consisting of a high dimensional feature space, however, such methods may be impractical or more difficult to apply to real data. Information-theoretic models of bounded rationality present an approach to dealing with decision problems where information is not fully available by including entropy or information-processing costs as part of the problem itself [16,17].

Recent work has broadened random-utility and discrete-choice models through heterogeneous or unobserved choice sets and preferences [18], limited consideration [19], feature-dependent mixtures learned from incomplete preferences [20], and gradient-boosted utility functions that retain a random-utility interpretation [21]. These studies primarily address the identification, estimation, or prediction of heterogeneous choice mechanisms. In contrast, our focus is on the distribution of aggregate market shares induced by a given population of preferences in a many-feature limit.

Other decision-making frameworks represent uncertainty at the level of structured individual assessments: pure fuzzy-soft sets encode interactions among assessment parameters [22], whereas neutrosophic fuzzy-soft similarity methods retain membership, indeterminacy, and non-membership information [23].

Consider a market, here defined as a population of individuals who choose a single option out of a collection of available alternatives. Each alternative is described by a set of numerical features. The behavior arising from such markets seems to typically fall somewhere between fully objective and fully subjective. When the perceived utility of an alternative is not affected too much by the individual making the decision, we say that the choice is objective. This can happen if choices depend mostly on one factor, for example, when all products perform similarly, but one is cheaper. On the other end of the spectrum, when the utility varies significantly among individuals, we say that the choice is subjective. For example, choices related to politics or art are inherently subjective—beauty is in the eye of the beholder.

Between these two extremes lies what we refer to as a competitive market. A competitive market is characterized by giving alternatives equal opportunity. This is not necessarily translated into equal market share. Instead, in competitive markets, pairwise contests between random alternatives are maximally unpredictable.

The main goal of this paper is to show that, if the dimension is large enough, then under mild assumptions, the statistics of the market follow a limiting behavior that can be analyzed without having to consider specific model features. Simulations are used to generalize the analytical results. We also demonstrate the applicability of the main results to classification algorithms.

The paper is organized as follows. Section 2 defines an infinite-supply preference market along with a linear realization of the multi-attribute utility model and compares it with empirical data studies. We then simulate the model to get a basic understanding of its behavior. Some definitions and specifics are deferred to Section 4, where we derive analytic formulas for the expected market share and its variance in a random market. We highlight a key factor that naturally emerges from the structure of the model—a measure of ’objectiveness’ versus ’subjectiveness’ in choice behavior. Section 5 shows that a softmax choice rule can be integrated into the model with minimal effect on its statistics. Finally, Section 6 demonstrates that our results may be applicable to a larger class of decision problems, including non-linear classification methods. We provide an example from the classification of random symbols using a neural network. Some details and proofs are deferred to Appendix A and Appendix B. Appendix C explores the way alternatives separate the space of individual weights into segments. Appendix D presents initial results for deriving formulae for the distribution of market shares in a random market. Appendix E discusses some extensions into non-linear utility (see also [24]).

## 2. Linear Preference Model

Consider a market consisting of *A* alternatives. Each alternative is characterized by *F* numerical features. The population consists of *I* individuals, each of whom decides on one of the alternatives by maximizing a personalized linear utility function, which is a weighted combination of the *F* features [4,5]. For simplicity, we assume the following:

1. Each alternative a=1…A is characterized by various measurable features. The measurement vector of an alternative contains the measured values of all features for that alternative and is denoted as ${\bar{m}}_{a}=\left(m_{a}^{1},\ldots ,m_{a}^{F}\right)\in R^{F}$.
2. Each individual i=1…I has a unique linear utility function, which assigns a real value to each alternative. Weight vectors are denoted as ${\bar{\omega }}_{i}=\left(\omega_{i}^{1},\ldots ,\omega_{i}^{F}\right)\in R^{F}$.
3. Information about the alternatives is complete. Every individual picks from the same pool of alternatives, using the same measurements. All individuals see the same measurements of all features. In particular, there is no missing or hidden information.
4. Individuals make their decision based only on the alternatives in the market, not based on choices of other individuals.
5. The perceived value (utility) of alternative *a* for individual *i* is,
  (1) $$v_{i,a}={\bar{\omega }}_{i}\cdot {\bar{m}}_{a}=\sum_{f=1}^{F}\omega_{i}^{f}m_{a}^{f}.\;$$
6. Each individual is assumed to choose the alternative with the highest value to them, i.e., ${arg max}_{a=1\ldots A}v_{i,a}$.
7. We assume independence across features and between weights and measurements. That is, for any $f\neq f^{\prime }$, $\omega_{i}^{f}$ and $\omega_{i}^{f^{\prime }}$ are independent, as well as $m_{a}^{f}$ and $m_{a}^{f^{\prime }}$, and any weight is independent of any measurement.

As an example, consider an individual seeking to purchase a vehicle and comparing multiple cars. Every car is described by various comparable numerical features such as price, speed, color, size, number of seats, running on gas/electricity, time between refueling/recharging, off-road capability, etc. Attributes such as price and gas consumption are relatively objective; however, a wealthy individual may assign a lower weight on costs, yet still care about the frequency of having to refuel. Other attributes may be subjective, as different individuals will look for different properties.

Due to the linearity of the model, certain situations are excluded from the model, such as those where the result is a non-linear mix of different components. In particular, while an individual may strongly favor two properties, they may not work well together, resulting in their combination being detrimental or causing diminishing returns. Such considerations cannot be modeled by a linear utility function. Additional linear model assumptions may not hold in some realistic scenarios [25]. For example, the assumption of full and coherent information on measurements, and on the existence of alternatives, is clearly non-trivial [26,27]. Recent heterogeneous-choice models allow the available or considered choice set itself to vary across individuals [18,19]. This source of variation is excluded from our base model and could otherwise be confounded with preference heterogeneity when only aggregate choices are observed. In some cases, such details can be taken into account using virtual measurements and weights. For example, adding an identity-specific feature that equals 1 for a specific alternative and 0 otherwise, with a large negative weight for an individual who does not know the alternative exists. Such approaches may lead to overfitting [28,29]. Finally, measurements and weights may be correlated. Some correlations may be reduced by principal component analysis.

The model is expected to be the most appropriate when choices aggregate many moderately important features, but may be less applicable if a single hard constraint dominates. We discuss this limitation further in Section 7.2.2.

### 2.1. Market Share

The market share of an alternative in a random market (i.e., random measurements and/or weights) is the average rate at which the alternative is chosen over the others. In other words, it is equal to the probability that a random individual *i* will pick alternative *a* out of all the available alternatives. There is an asymmetry within the structure of markets: when the number of individuals is large enough, due to the law of large numbers, adding more individuals does not change market shares as long as the underlying weight distributions remain the same. Adding new alternatives, on the other hand, adds competition to the existing alternatives, which reduces their total shares.

Often we will be interested in the market share of an alternative in a market where the rest of the alternatives are unknown. In such a case, the market share of alternative *a* can be defined as a random variable that, in the limit of I→∞, approaches the probability

(2) $$T_{a}\left({\bar{m}}_{a},M_{a}\right)=P_{{\bar{\omega }}_{i}}\left(v_{i,a}>v_{i,a^{\prime }},\;\forall a^{\prime }\neq a\;|\;{\bar{m}}_{a}\right).$$

Here, $M_{a}$ is a matrix in which the columns are the measurement vectors from the market, aside from ${\bar{m}}_{a}$. In this notation, we think of ${\bar{m}}_{a}$ as a fixed vector, i.e., the measurements of alternative *a* are known, but $M_{a}$ as random, i.e., the other measurements are random.

### 2.2. Market Types

To quantify the distinction between objective and subjective markets, we compare two components of the population’s preferences. The first is the shared component: the average direction in feature space toward which individuals tend to agree. It is given by the mean of a random weight vector, denoted as ${\bar{\mu }}_{W}$. The second is the heterogeneity component: the amount by which individual preferences vary around this average direction, and is given by the standard deviation of a weight vector, denoted as ${\bar{\sigma }}_{W}$.

**Definition** **1.** *Degree of subjectivity. The market’s degree of subjectivity is defined as $\eta :=\|{\bar{\sigma }}_{W}\|/\|{\bar{\mu }}_{W}\|$. It ranges between zero for a fully objective market and infinity for a fully subjective market. It serves as a multivariate analogue to a coefficient of variation.*

Intuitively, $\left\|{\bar{\sigma }}_{W}\|\ll \|{\bar{\mu }}_{W}\right\|$ implies that there are a few features that dominate the selection, and that variance in preference is limited. Here, individuals mostly look for the same thing in an alternative, i.e., the market is objective. On the other hand, if $\left\|{\bar{\sigma }}_{W}\|\gg \|{\bar{\mu }}_{W}\right\|$, the variance is large compared to the mean, and it is expected that every individual will choose differently. Accordingly, we call the market subjective. The special case η=1 will be shown to correspond to a competitive market, in which pairwise contests between random alternatives are maximally unpredictable. The surprising result is that as I,F→∞, the characterization of markets depends only on η.

To illustrate the meaning of the degree of subjectivity, consider the following analogy (equivalently, one may regard the varying daily conditions as playing the role of different realizations of individual preference). We come across a local chess club and learn that upsets do not happen frequently relative to the players’ Elo scores. If we pick two players at random to play many games, we can expect one of the pair to win most matches. This club corresponds to an objective market. In another club, a different chess opening is imposed each day, and different players specialize in different openings. If we pick two competitors at random, then on any given day one player may dominate their matches, but which player dominates depends strongly on the imposed opening. Across many randomly selected days, neither player has a systematic advantage. Such a club corresponds to a subjective market. Now consider a third club, somewhere between those two extremes. After picking two competitors at random, we observe their matches against one another over many randomly selected days. In this club, however, the eventual split of wins is maximally unpredictable. The proportion of games won by either player is uniformly distributed between 0 and 1 (for simplicity of the analogy, we ignore draws). Such a club reflects the maximal-entropy condition of a competitive market.

**Definition** **2.** *Comparison distribution. Consider a simple market with only two alternatives, A=2. We refer to the distribution of the market share as a comparison distribution. As we will show later in the paper, this distribution reaches a stable limit as F→∞ of the form*

(3) $$P\left(T_{a}<t\right)=\Phi \left(\eta \;\Phi^{-1}\left(t\right)\right),$$

*where* Φ *is the cumulative distribution function (CDF) of the standard normal distribution.*

**Definition** **3.** *Comparison entropy. The comparison entropy of a market with A=2 is defined as the differential entropy of its comparison distribution.*

(4) $$H=-E[\log c_{\eta }\left(T_{1}\right)],$$

*where $c_{\eta }$ is the probability density function (PDF) of the comparison distribution for a market with degree of subjectivity η. Note that the comparison distribution is symmetric around 1/2, and there is no difference between choosing $T_{1}$ or $T_{2}$ here.*

**Definition** **4.** *Competitive market. A market is said to be competitive when its comparison entropy is maximal, which implies maximal uncertainty when comparing two alternatives at random.*

In Section 4.6, we prove Equation (3) and show that in the limit I,F→∞, the comparison entropy depends only on η, i.e., the degree of subjectivity of the market. Moreover, a market is competitive exactly when η=1. In this paper, we do not make any claims about the connection between competitiveness in a preference market and existing measures of competitiveness in economic theory. It is not clear how, or in what capacity, being competitive affects a preference market—for example, if time dynamics or pricing were introduced to the model. We leave the reader only with the maximal-entropy condition as intuition behind the name.

Note that the definition for comparison distribution can be generalized to A>2 by averaging the comparison distributions of all alternative pairs, thus enabling the same empirical analysis for larger markets.

## 3. Empirical Data and Simulations

In this section, we compare empirical data with simulations of the linear preference model. We do this in three ways. First, we demonstrate the behavior of simulated markets based on varying parameters, as well as different distribution types used for weights and measurements. Turning to real markets, we compare simulations of the linear model with example empirical market share data. Lastly, we look at empirical comparison distributions generated from full-ranking information, which is richer than knowing only the individual’s best alternative. The empirical results are summarized and sorted by comparison entropy in Table 1. Our goal is to demonstrate the applicability of the degree of subjectivity parameter η in describing the full market share distribution in real markets.

Simulating the preference decision process provides an intuitive understanding of the model’s output and its implications. By generating random weights, $W\in R^{F\times I}$, and measurements, $M\in R^{F\times A}$, the product $W^{T}M$ determines the empirical market share $T_{a}\left(W,M\right)$, which can be used as an approximation for the non-empirical version when using a large value for *I*.

**Figure 1:**
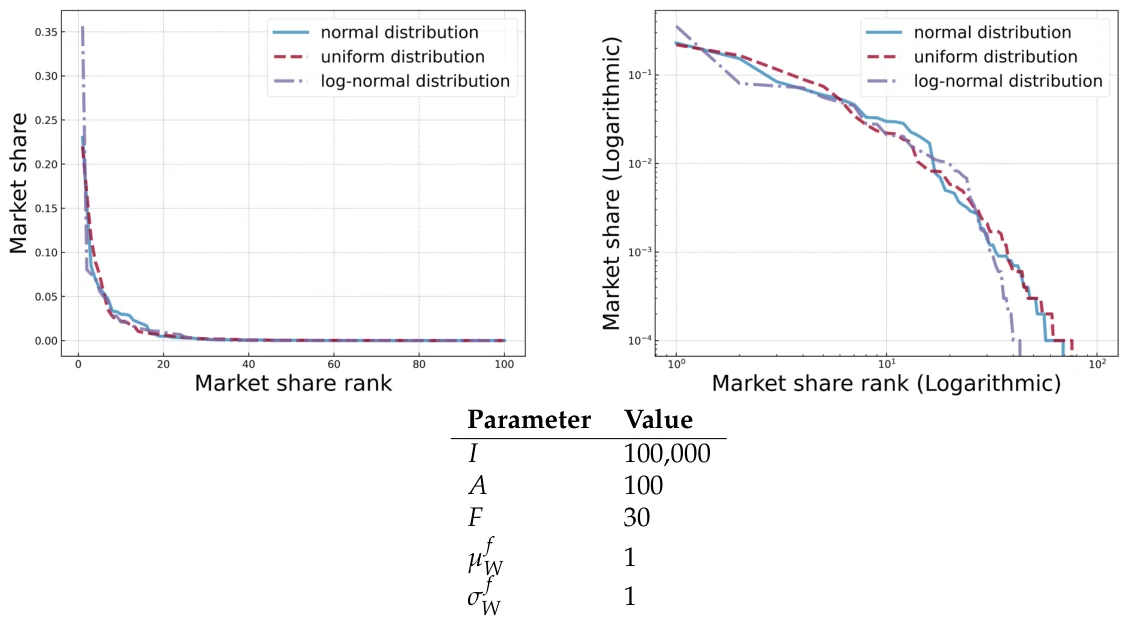
Simulated random markets with uniform, normal, and log-normal distributions for both weights and measurements. (**Left**): a rank-frequency distribution corresponding to markets generated by a different distribution, with identical means and variances. (**Right**): the same data on a log–log scale.

Figure 1 shows the rank-frequency graphs (which are also the empirical distributions of market share) of preference markets generated via different types of distributions. As shown below in Section 4.2, we can set $\mu_{M}^{f}=0$ and $\sigma_{M}^{f}=1$ for all *f*. For simplicity, we use the same distribution for all weights and the same distribution for all measurements. The numerical results demonstrate the applicability of a central limit argument, establishing that when the number of features *F* is large, the market share distribution does not depend on the particular choice of distribution. Accordingly, for the rest of the paper simulations we will be using normal distributions for both weights and measurements.

**Figure 2:**
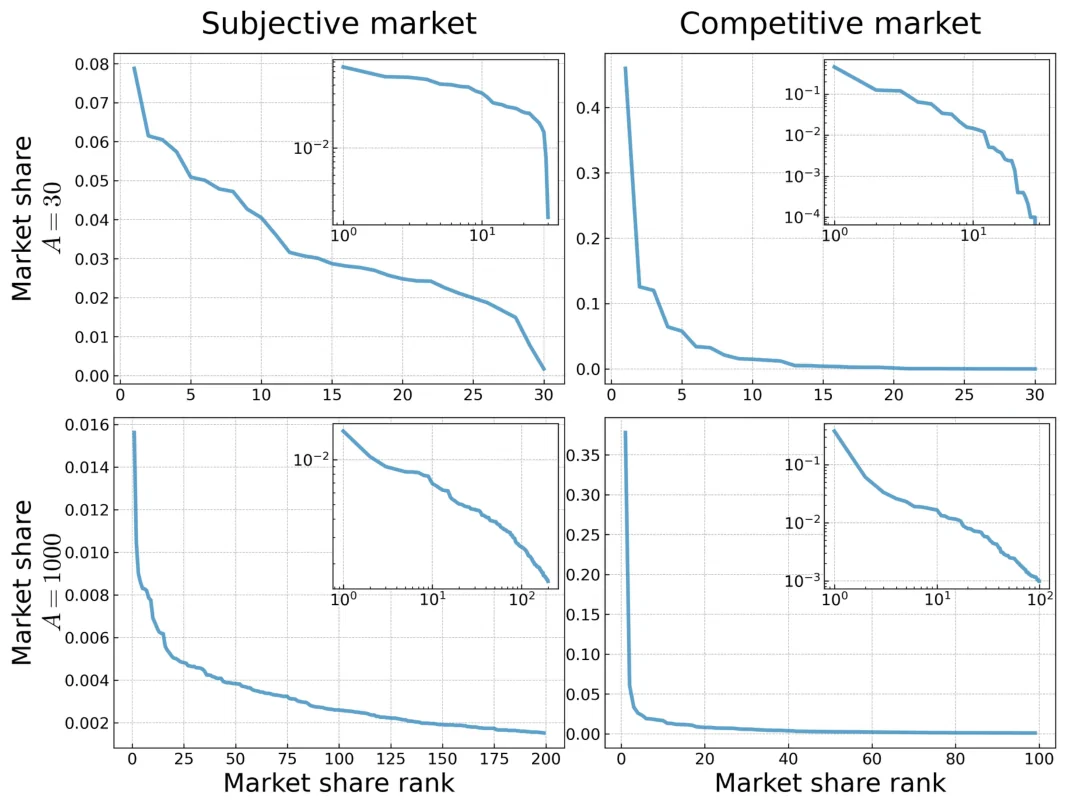
Rank-frequency plots for preference markets generated by simulation. The (**first row**) shows small markets at size A=30, while the (**second row**) shows larger markets with A=1000, showing only the top alternatives. (**Left column**): subjective markets, generated by setting η=∞. (**Right column**): competitive markets, generated by setting η=1. Smaller subjective markets have a distinct curvy shape, quite unlike their more objective counterparts.

Next, we analyze the distinction between objective and subjective decision problems within the model. A subjective problem can be thought of as one in which, for each individual who likes an aspect or element, there exists another individual who dislikes it as much. In terms of the model, we set $\mu_{W}^{f}=0$ for each *f*. Figure 2 shows the market share rank-frequency distribution that emerges in subjective and competitive markets, with varying market sizes. In subjective markets, the structure of rank-frequency distributions changes as the number of alternatives decreases, forcing individuals to select less preferred options. Conversely, when $\mu_{W}^{f}\gg \sigma_{W}^{f}$ for all *f*, individual preferences converge to a shared value function $\sum_{f=1}^{F}\mu_{W}^{f}m_{a}^{f}$ which would give rise to a single alternative getting the lion’s share of the market. In between these two extremes, we get a mixed decision problem with varying degrees of subjectivity. In contrast to subjective markets, markets that are more objective appear to retain their characteristic shape, even as the number of available alternatives decreases. When $\eta =\|{\bar{\sigma }}_{W}\|/\|{\bar{\mu }}_{W}\|=1$, the market is competitive, which means the comparison entropy is maximized (see Section 4.6).

Finally, we turn to study empirical examples of real markets. Finding static preference markets is challenging, as many real markets undergo certain dynamics that could influence the distribution of the measurement vectors in share-aware ways, which may not be possible to account for by a change in η alone.

**Figure 3:**
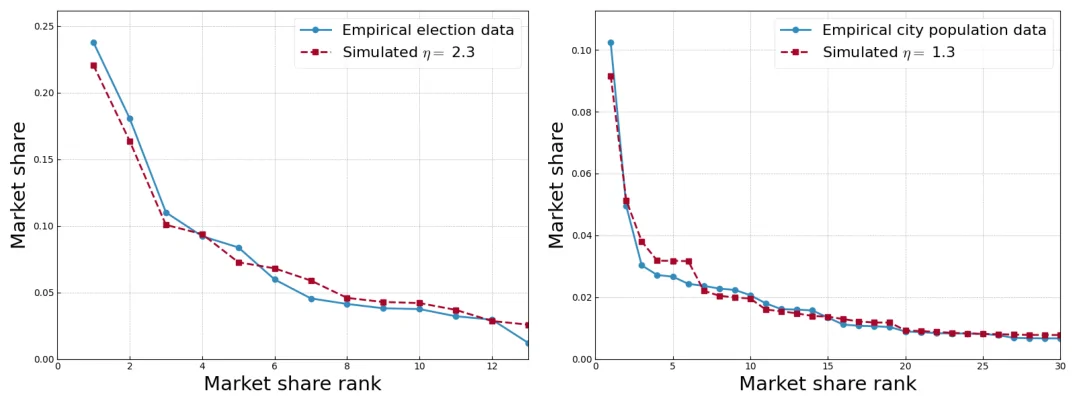
(**Left**): Israeli 2022 legislative election results (election data taken from https://votes25.bechirot.gov.il/ (accessed on 5 September 2025)) (blue), compared to a simulated random market with η=2.3 (red). Higher η indicates a more subjective market. (**Right**): Israeli city population data (Population data taken from the Israeli Central Bureau of Statistics at https://www.cbs.gov.il/en (accessed on 5 September 2025)) from 2022 (blue, top 100 shown only), compared to a simulated random market with η=1.3 (red).

Figure 3 shows comparisons of the rank frequency plots generated from population and election data in Israel, with the rank frequency plots of randomly generated preference markets using different degrees of subjectivity for each, fitted to the data by ad-hoc means of generating random markets using various values of η.

**Figure 4:**
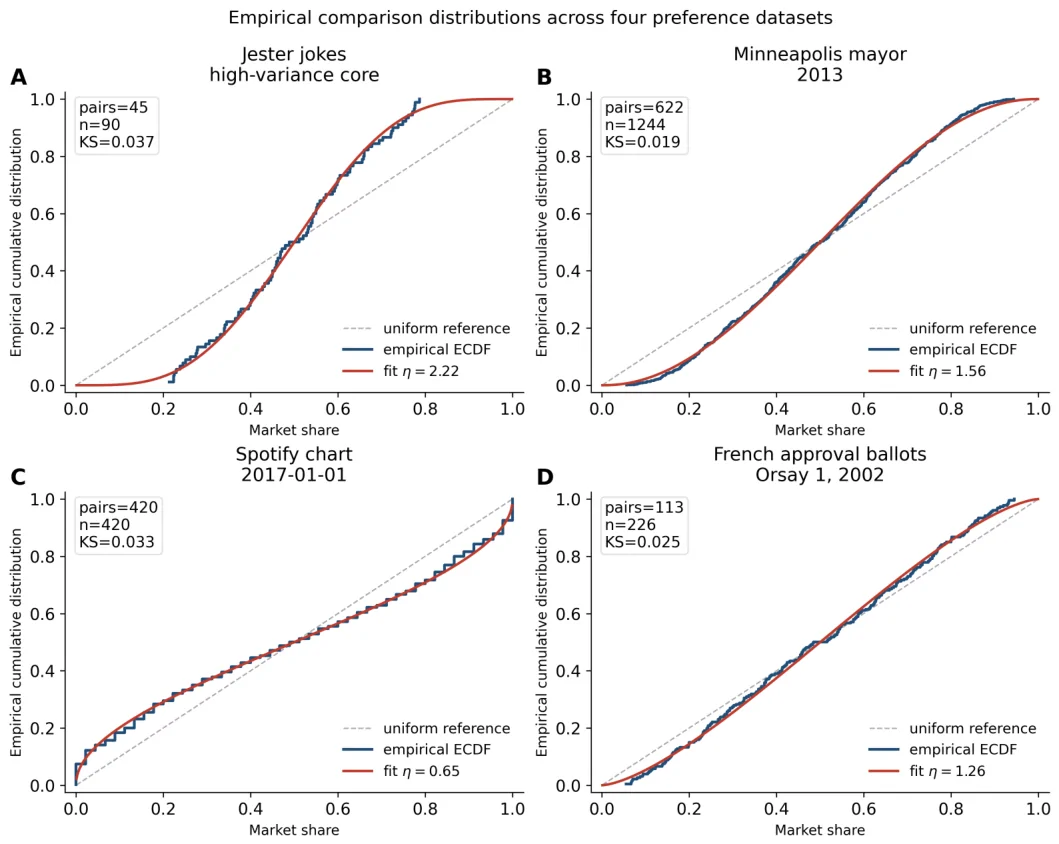
Empirical comparison distributions formed by counting ratios of population splits in pairwise comparisons. The values of η were fit using a standard minimum Kolmogorov–Smirnov estimator. (**A**) Pairwise comparisons of joke rankings, including negative values. Part of the Jester data set [30] (https://goldberg.berkeley.edu/jester-data/ (accessed on 5 September 2025)). (**B**) Minneapolis mayoral elections from 2013, where a total of 35 candidates were voted for by ranked choice, (https://dataverse.harvard.edu/file.xhtml?fileId=11593264&version=25.0 (accessed on 5 September 2025)). (**C**) Elections generated by querying the 200 most listened to songs on Spotify, using their particular ratings in individual countries as a proxy for individual choosers (Preflib [31] dataset number 00047). (**D**) An experiment [32] conducted during the French presidential election of 2002, where voters engaged in approval voting could mark any number of candidates as a binary yes/no. For the purpose of comparison distribution, only cases where an individual marked one candidate as yes and another as no were taken into account (Preflib dataset number 00026).

Another source of data, which is more informative and sometimes easier to collect, comes from pairwise comparison polls. Figure 4 shows real data collected from online polls of various origins, alongside the approximate market share distribution in Equation (3). Higher values of η imply more subjective markets, while lower values are tied to objective markets.

## 4. Analytical Results

In this section, we derive the main analytical results. In particular, we provide a formula for the expected market share of an alternative in a random market.

### 4.1. Definitions

We start by properly defining the objects of study: preference markets and market shares.

**Definition** **5.** *Random market. We define a market as a pair (W,M), where $W\in R^{F\times I}$ and $M\in R^{F\times A}$ are random matrices containing all weights and measurements, respectively:*

(5) $$W=\left(\begin{array}{ccc} | & \; & |\\ {\bar{\omega }}_{1} & \ldots & {\bar{\omega }}_{I}\\ | & \; & | \end{array}\right),\;M=\left(\begin{array}{ccc} | & \; & |\\ {\bar{m}}_{1} & \ldots & {\bar{m}}_{A}\\ | & \; & | \end{array}\right).$$

*The distribution of weight vectors is denoted as $D_{W}$, and we assume that the individual weights are independent and identically distributed (IID). The marginal distributions are denoted $\omega_{i}^{f}∼D_{W}^{f}$, having finite means and standard deviations $\mu_{W}^{f}$ and $\sigma_{W}^{f}$, respectively, for each f. Note that weights may be negative and do not necessarily sum to 1. The distribution of measurements is denoted by $D_{M}$, and we assume that the measurement vectors of different alternatives are IID. The marginal distributions are denoted by $m_{a}^{f}∼D_{M}^{f}$, having finite means and standard deviations $\mu_{M}^{f}$ and $\sigma_{M}^{f}$, respectively, for each f.*

*Furthermore, we denote the domains of $D_{W}$ and $D_{M}$ by $\Omega_{W}$ and $\Omega_{M}$, respectively. The distributions are both absolutely continuous with respect to the Lebesgue measures $dP({\bar{\omega }}_{i})$ and $dP({\bar{m}}_{a})$, respectively. Notice that these are distributions for a single vector, not a matrix (because they do not depend on i and a).*

In addition to finite second moments, our high-dimensional market share approximations will require assuming some further mild conditions on higher moments, which roughly translate to ensuring that no single feature is solely responsible for a large portion of the decisions made. The model itself is defined regardless of these additional conditions, though it may appear more fruitful when they hold. For specifics, see Theorems A3 and A4, as well as the discussion in Section 7.2.2.

**Definition** **6.** *Realized market. A realized market is a realization of M alone, but not W, i.e., W is still random. In other words, we know all the products in the market and their features. However, we do not know the particular individuals making their choices, only their statistics. We denote a realized market by M.*

Below, we will also consider markets in which only one alternative, *a*, is realized, but not the rest of them (i.e., the measurements of all other alternatives are still random). For that, we may use a minor matrix, $M_{a}$, which has alternative *a* omitted,

(6) $$M_{a}:=\left(\begin{array}{cccccc} | & \; & | & | & \; & |\\ {\bar{m}}_{1} & \ldots & {\bar{m}}_{a-1} & {\bar{m}}_{a+1} & \ldots & {\bar{m}}_{A}\\ | & \; & | & | & \; & | \end{array}\right).$$

**Definition** **7.** *Choice indicator. The choice indicator is a random variable defined as*

(7) $$c_{i,a}\left(W,M\right)=\left\{\begin{array}{cc} 1 & v_{i,a}>v_{i,a^{\prime }}\;\forall a^{\prime }\neq a\\ 0 & \mathrm{otherwise}. \end{array}\right.$$

In other words, $c_{i,a}=1$ if alternative *a* is the best choice for individual *i*. The choice indicator is dependent both on individual *i*’s weights, ${\bar{\omega }}_{i}$, and on the measurements of the entire market M. We assume that $\sum_{a=1}^{A}c_{i,a}=1$ with probability 1, meaning there are no ties. This limits us to markets with at least one continuous measurement, but we may still apply the model if the probability of a tie is negligible.

**Definition** **8.** *Market Share. The market share of alternative a in a random market (W,M) is the average rate at which the alternative is chosen over the others. In other words, it is equal to the probability that a random individual i will pick a out of all alternatives.*

By realizing the weights as well as the measurements M=M,W=W, we can define the empirical market share of alternative *a* as

(8) $$T_{a}\left(W,M\right):=\frac{1}{I}\sum_{i=1}^{I}c_{i,a}\left(W,M\right).$$

As the number of individuals *I* becomes large, the law of large numbers (LLN) applies, and using realized weights as opposed to a random variable becomes unnecessary. The (non-empirical) market share of alternative *a* is then provided by

(9) $$T_{a}\left(M\right):=E_{W}\left[T_{a}\left(W,M\right)\right]=\lim_{I\to \infty }\frac{1}{I}\sum_{i=1}^{I}c_{i,a}=P_{{\bar{\omega }}_{i}}\left(v_{i,a}>v_{i,a^{\prime }},\;\forall a^{\prime }\neq a\;|\;M\right).$$

Here, $P_{X}\left(\cdot \right)$ and $E_{X}\left[\cdot \right]$ denote the probability and expectation with respect to a random variable or vector *X*, respectively. Notice that in (9), the left-hand side (LHS) does not depend on *i*, while the right-hand side (RHS) does. This is because ${\bar{\omega }}_{i}$ are IID.

There is an asymmetry within the structure of markets: when the number of individuals is large enough, adding more individuals (and, thus, weight vectors) does not change market shares, as long as the underlying weight distributions remain the same. Adding new alternatives, on the other hand (and, therefore, new measurement vectors), adds competition to existing alternatives.

For a random market, the market share of a single realized alternative *a* in an otherwise random market is a random variable dependent on $M_{a}$,

(10) $$T_{a}\left({\bar{m}}_{a},M_{a}\right)=P_{{\bar{\omega }}_{i}}\left(v_{i,a}>v_{i,a^{\prime }},\;\forall a^{\prime }\neq a\;|\;{\bar{m}}_{a}\right).$$

In this notation, we think of ${\bar{m}}_{a}$ as a fixed vector, i.e., the measurements of alternative *a* are known, but $M_{a}$ is random, i.e., the other measurements are random.

### 4.2. Standardizing the Model Parameters

In order to reduce the degrees of freedom when defining a random market, we define and single out the preference order, which solely determines the market share.

**Definition** **9.** *Preference order. The preference order of a realized market (W,M) is a distribution over permutation of length A, given by $\pi_{\sigma }=P_{{\bar{\omega }}_{i}}\left(v_{i,\sigma_{1}}>v_{i,\sigma_{2}}>\cdots >v_{i,\sigma_{A}}\right)$ for every permutation $\sigma \in S_{A}$.*

**Remark** **1.** *The marginal distributions, and in turn the preference order, fully determine market shares. Any change in the market that leaves the preference order unchanged will also leave market shares unchanged. This allows us to derive a standardized form for a market.*

There are certain transformations one can apply to both weight and measurement vectors without changing the market’s preference order. For example, by multiplying either each weight $\omega_{i}^{f}$ or each measurement $m_{a}^{f}$ by a constant C>0, or by multiplying all weights of a specific feature $\omega_{i}^{f}$ by a constant and dividing each corresponding measurement $m_{a}^{f}$ by the same constant.

An interesting asymmetry arises when one tries to add a constant value to a specific feature’s weight or measure:

(11) $$v_{i,a}^{\prime }=\sum_{f=1}^{F}\left(\omega_{i}^{f}+C\right)m_{a}^{f}=v_{i,a}+C\sum_{f=1}^{F}m_{a}^{f},$$

(12) $$v_{i,a}^{\prime }=\sum_{f=1}^{F}\omega_{i}^{f}\left(m_{a}^{f}+C\right)=v_{i,a}+C\sum_{f=1}^{F}\omega_{i}^{f}.$$

Since the first case depends on the alternative’s measurements ${\bar{m}}_{a}$, it could alter the preference order because it affects each alternative differently. The second case, however, depends only on the individual and therefore adds the same bias to the utility of all alternatives. Therefore, it does not affect the preference order.

Using these degrees of freedom, we can shift the distributions $D_{W}^{f}$ and $D_{M}^{f}$ such that $\mu_{M}^{f}=E\left[m_{a}^{f}\right]=0$ and ${\sigma_{M}^{f}}^{2}=E\left[{\left(m_{a}^{f}\right)}^{2}\right]=1$. This leaves us with one other degree of freedom, which we can use to normalize each weight vector. While this does not affect the preference order, it results in some loss of information on the relative contribution of features. Since, in this paper, we are mostly interested in the market share, unless stated explicitly, we will be working with normalized weights, $\|{\bar{\omega }}_{i}\|=1$.

Due to the parameter standardization, we can write

(13) $$\|{\bar{\mu }}_{W}\|=\frac{1}{\sqrt{1+\eta^{2}}},\;\|{\bar{\sigma }}_{W}\|=\frac{\eta }{\sqrt{1+\eta^{2}}}.\;$$

### 4.3. Expected Market Share

We proceed to calculate the expected market share of a specific alternative in a random market.

Recall that the random variable $M_{a}$ is a minor of the measurement matrix M created by omitting the column containing ${\bar{m}}_{a}$. As in Equation (10), we have

(14) $$T_{a}\left({\bar{m}}_{a},M_{a}\right)=P_{\;{\bar{\omega }}_{i}}\left(v_{i,a}>v_{i,a^{\prime }}\;\forall a^{\prime }\neq a\;|\;M\right).\;$$

If we condition over ${\bar{m}}_{a}$ and take expectation over $M_{a}$, we are left with a random variable over ${\bar{\omega }}_{i}$:

(15) $$\begin{array}{cc} E_{M_{a}}\left[T_{a}|\;{\bar{m}}_{a}\right] & =\int_{\Omega_{M}^{A-1}}P_{{\bar{\omega }}_{i}}\left({\bar{\omega }}_{i}\cdot {\bar{m}}_{a}>{\bar{\omega }}_{i}\cdot {\bar{m}}_{a^{\prime }}\;\forall a^{\prime }\neq a\;|\;M\right)\;dP\left(M_{a}\right)\\ & =\int_{\Omega_{M}^{A-1}}\int_{\Omega_{W}}c_{i,a}\;dP\left({\bar{\omega }}_{i}\right)\;dP\left(M_{a}\right).\; \end{array}$$

Since the integrand is bounded, we can switch the order of integration as follows:

(16) $$\int_{\Omega_{W}}\int_{\Omega_{M}^{A-1}}c_{i,a}\;dP\left(M_{a}\right)\;dP\left({\bar{\omega }}_{i}\right)=\int_{\Omega_{W}}P_{M_{a}}\left({\bar{\omega }}_{i}\cdot {\bar{m}}_{a}>{\bar{\omega }}_{i}\cdot {\bar{m}}_{a^{\prime }}\;\forall a^{\prime }\neq a\;|\;{\bar{\omega }}_{i},\;{\bar{m}}_{a}\right)\;dP\left({\bar{\omega }}_{i}\right).$$

Since ${\bar{m}}_{a^{\prime }}$ are independent, this expression can be simplified to

(17) $$\int_{\Omega_{W}}P_{{\bar{m}}_{a^{\prime }}}{\left({\bar{\omega }}_{i}\cdot {\bar{m}}_{a}>{\bar{\omega }}_{i}\cdot {\bar{m}}_{a^{\prime }}\;|\;{\bar{\omega }}_{i},\;{\bar{m}}_{a}\right)}^{A-1}\;dP\left({\bar{\omega }}_{i}\right).$$

Inside the parentheses we have *F* random variables $\omega_{i}^{1}m_{a^{\prime }}^{1}\ldots \omega_{i}^{F}m_{a^{\prime }}^{F}$, each with mean 0 and variance ${\left(\omega_{i}^{f}\right)}^{2}<\infty$. Under some mild conditions, the sum converges to a normal random variable. However, because we are looking to integrate over $\Omega_{W}$, the error might diminish impractically slowly. In order to bound this error, we need to show uniform convergence of ${\bar{\omega }}_{i}\cdot {\bar{m}}_{a^{\prime }}$ over $\Omega_{W}$, which we can achieve by employing the Berry–Esseen theorem. The full statement and proof can be found in Appendix B; here, we only state the following version, which we need in order to derive the expected value.

**Theorem** **1.** *Assume that $\sum_{f=1}^{F}E[|\omega_{i}^{f}{|}^{3}]\cdot E[|m_{a^{\prime }}^{f}{|}^{3}]\underset{F\to \infty }{\to }0$ and $\sum_{f=1}^{F}E\left[{\left(\omega_{i}^{f}\right)}^{4}\right]\underset{F\to \infty }{\to }0$. Then, we have*

(18) $$\lim_{F\to \infty }\left|\int_{\Omega_{W}}P_{{\bar{m}}_{a^{\prime }}}{\left({\bar{\omega }}_{i}\cdot {\bar{m}}_{a^{\prime }}<{\bar{\omega }}_{i}\cdot {\bar{m}}_{a}\;|\;{\bar{\omega }}_{i}\right)}^{A-1}dP\left({\bar{\omega }}_{i}\right)-\int_{\Omega_{W}}\Phi {\left({\bar{\omega }}_{i}\cdot {\bar{m}}_{a}\right)}^{A-1}dP\left({\bar{\omega }}_{i}\right)\right|=0,$$

*where* Φ *denotes the cumulative distribution function (CDF) of the standard normal distribution.*

We now obtain

(19) $$\lim_{F\to \infty }\int_{\Omega_{W}}P_{{\bar{m}}_{a^{\prime }}}{\left({\bar{\omega }}_{i}\cdot {\bar{m}}_{a}>{\bar{\omega }}_{i}\cdot {\bar{m}}_{a^{\prime }}\;|\;{\bar{\omega }}_{i},\;{\bar{m}}_{a}\right)}^{A-1}\;dP\left({\bar{\omega }}_{i}\right)=\int_{\Omega_{W}}\Phi {\left({\bar{\omega }}_{i}\cdot {\bar{m}}_{a}\right)}^{A-1}\;dP\left({\bar{\omega }}_{i}\right).$$

We observe that the element within the CDF is yet another random vector dot product:

(20) $${\bar{\omega }}_{i}\cdot {\bar{m}}_{a}\underset{F\to \infty }{\overset{d}{\to }}N\left({\bar{\mu }}_{W}\cdot {\bar{m}}_{a},{\bar{\sigma }}_{W}^{2}\cdot {\bar{m}}_{a}^{2}\right),$$

where ${\bar{\sigma }}_{W}^{2}$ and ${\bar{m}}_{a}^{2}$ are provided by element-wise squaring of the original vector components. A proof of convergence in distribution can be found in Theorem A4. Assuming that $\sqrt{{\bar{\sigma }}_{W}^{2}\cdot {\bar{m}}_{a}^{2}}$ does not vanish, we can change variables to obtain

(21) $$E_{M_{a}}\left[T_{a}|\;{\bar{m}}_{a}\right]\approx \frac{1}{\sqrt{{\bar{\sigma }}_{W}^{2}\cdot {\bar{m}}_{a}^{2}}}\int_{-\infty }^{\infty }\Phi {\left(x\right)}^{A-1}\phi \left(\frac{x-{\bar{\mu }}_{W}\cdot {\bar{m}}_{a}}{\sqrt{{\bar{\sigma }}_{W}^{2}\cdot {\bar{m}}_{a}^{2}}}\right)\;dx.$$

**Figure 5:**
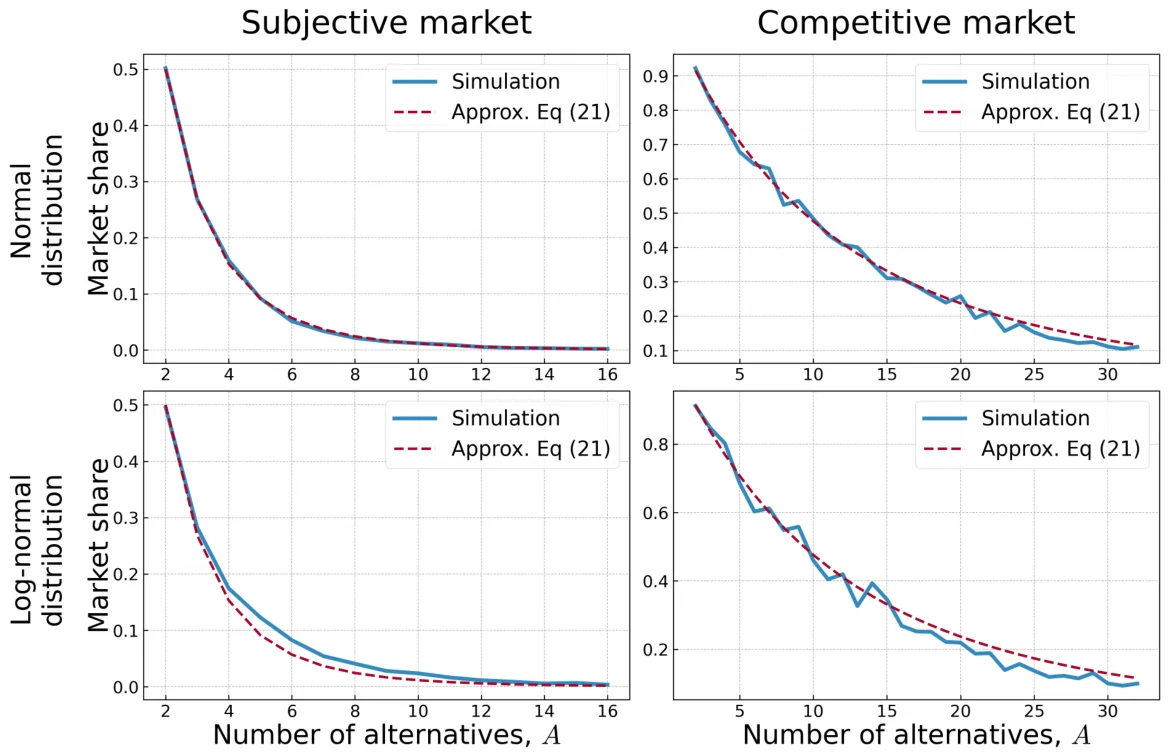
Comparison of the derived integral estimate of the expected market share and simulated data using normal distributions (**top row**) or log-normal distributions (**lower row**) for weights and measurements. (**Left**) Subjective market with η=∞. (**Right**) Competitive market with η=1. In both cases, we set $F=30,\;m_{a}^{f}=2/\sqrt{F}$. In the log-normal case, the heavier tail in the distribution can cause the approximation to converge much slower.

We leave to Section 7.2.2 the discussion of the practical implications of the moment assumptions we had to take for Theorem 1. Equation (A8) also provides a finite-dimensional diagnostic for the first Gaussian approximation. In our notation, its error is controlled by the term $\sum_{f=1}^{F}E[|\omega_{i}^{f}{|}^{3}]E[|m_{a^{\prime }}^{f}{|}^{3}]$, together with the concentration of the weight-vector norm established in the proof. For moderate values of *F*, the error therefore depends on how quickly this term tends to zero. Figure 5 shows a comparison between the estimate and simulations. Here, we see our first motivation for normalizing the weights when we are only interested in the market share. Setting $\|{\bar{\omega }}_{i}\|=1$ does not change the preference order, and makes the use of A4 and A3 cleaner.

### 4.4. Market Share Variance

We next consider the *L*’th moment of $T_{a}\left({\bar{m}}_{a},M_{a}\right)$.

(22) $$\begin{array}{cc} E_{M_{a}}\left[T_{a}^{L}|\;{\bar{m}}_{a}\right] & =\int_{\Omega_{M}^{A-1}}P_{{\bar{\omega }}_{i}}{\left({\bar{\omega }}_{i}\cdot {\bar{m}}_{a}>{\bar{\omega }}_{i}\cdot {\bar{m}}_{a^{\prime }}\;\forall a^{\prime }\neq a\;|\;M\right)}^{L}\;dP\left(M_{a}\right)\\ & =\int_{\Omega_{M}^{A-1}}{\left(\;\int_{\Omega_{W}}c_{i,a}\;dP\left({\bar{\omega }}_{i}\right)\right)}^{L}\;dP\left(M_{a}\right). \end{array}$$

In order to switch the order of integration, we expand the power, each with an independent random weight vector:

(23) $$\int_{\Omega_{W}^{L}}\int_{\Omega_{M}^{A-1}}\prod_{l=1}^{L}1_{\left\{{\bar{\omega }}_{l}\cdot {\bar{m}}_{a}>{\bar{\omega }}_{l}\cdot {\bar{m}}_{a^{\prime }}\;\forall a^{\prime }\neq a\right\}}\left({\bar{m}}_{a},M_{a}\right)\;dP\left(M_{a}\right)\;\prod_{l=1}^{L}dP\left({\bar{\omega }}_{l}\right).$$

Each inner product ${\bar{\omega }}_{l}\cdot {\bar{m}}_{a^{\prime }}$ is again approximately normally distributed. However, unlike the L=1 case, they are now dependent. As a result, we need to consider them together as a multivariate normal distribution. By replacing the order of integration and integrating over $dP(M_{a})$, we obtain

(24) $$\int_{\Omega_{W}^{L}}P_{\bar{m}}{\left({\bar{\omega }}_{l}\cdot {\bar{m}}_{a}>{\bar{\omega }}_{l}\cdot \bar{m},\;\forall 1\leq l\leq L\;|\;{\bar{m}}_{a}\right)}^{A-1}\;dP\left(W\right),$$

where

(25) $$W=\left(\begin{array}{ccc} | & \; & |\\ {\bar{\omega }}_{1} & \ldots & {\bar{\omega }}_{L}\\ | & \; & | \end{array}\right)\in R^{F\times L}.$$

Define $x_{l}:={\bar{\omega }}_{l}\cdot \left(\bar{m}-{\bar{m}}_{a}\right),\;\bar{x}=\left(x_{1},\ldots ,x_{L}\right)$. Recall that $E[m_{a}^{f}]=0$ for all *f*. As F→∞, $\bar{x}\overset{d}{\to }N\left(-W^{T}{\bar{m}}_{a},W^{T}W\right)$. We can now write the *L*’th moment as

(26) $$\int_{\Omega_{W}^{L}}{\left(\int_{R_{-}^{L}}\frac{\exp(-\frac{1}{2}{\left(\bar{x}+W^{T}{\bar{m}}_{a}\right)}^{T}{\left(W^{T}W\right)}^{-1}\left(\bar{x}+W^{T}{\bar{m}}_{a}\right))}{\sqrt{{\left(2\pi \right)}^{L}\left|W^{T}W\right|}}\;d\bar{x}\right)}^{A-1}dP\left(W\right).$$

This integral is difficult to evaluate in general. We proceed to calculate the second moment of the market share, which encompasses information about the risk of inserting a known alternative into an unknown market. Observe that, given mild conditions, each product ${\bar{\omega }}_{l}\cdot {\bar{\omega }}_{l^{\prime }}$ is normal as well. Particularly, for the second moment, L=2, we have

(27) $$\left(\begin{array}{c} z_{1}\\ z_{2}\\ z_{3} \end{array}\right):=\left(\begin{array}{c} {\bar{\omega }}_{1}\cdot {\bar{m}}_{a}\\ {\bar{\omega }}_{2}\cdot {\bar{m}}_{a}\\ {\bar{\omega }}_{1}\cdot {\bar{\omega }}_{2} \end{array}\right)∼N\left({\bar{\mu }}_{2},\Sigma_{2}\right),$$

(28) $${\bar{\mu }}_{2}=\left(\begin{array}{c} {\bar{\mu }}_{W}\cdot {\bar{m}}_{a}\\ {\bar{\mu }}_{W}\cdot {\bar{m}}_{a}\\ {\left\|{\bar{\mu }}_{W}\right\|}^{2} \end{array}\right),\;\Sigma_{2}=\left(\begin{array}{ccc} {\bar{\sigma }}_{W}^{2}\cdot {\bar{m}}_{a}^{2} & 0 & {\bar{\mu }}_{W}\cdot \left({\bar{m}}_{a}⊙{\bar{\sigma }}_{W}^{2}\right)\\ 0 & {\bar{\sigma }}_{W}^{2}\cdot {\bar{m}}_{a}^{2} & {\bar{\mu }}_{W}\cdot \left({\bar{m}}_{a}⊙{\bar{\sigma }}_{W}^{2}\right)\\ {\bar{\mu }}_{W}\cdot \left({\bar{m}}_{a}⊙{\bar{\sigma }}_{W}^{2}\right) & {\bar{\mu }}_{W}\cdot \left({\bar{m}}_{a}⊙{\bar{\sigma }}_{W}^{2}\right) & {\left\|{\bar{\sigma }}_{W}^{2}\right\|}^{2}+2{\bar{\mu }}_{W}^{2}\cdot {\bar{\sigma }}_{W}^{2} \end{array}\right),$$

where ⊙ denotes element-wise vector product. By normalizing the weights, we obtain

(29) $$W^{T}W=\left(\begin{array}{cc} 1 & z_{3}\\ z_{3} & 1 \end{array}\right).$$

Substituting $\bar{u}=\bar{x}+W^{T}{\bar{m}}_{a}$, the inner integral in (26) becomes

(30) $$\Phi_{2}\left(z_{1},z_{2},z_{3}\right):=\int_{-\infty }^{z_{1}}\int_{-\infty }^{z_{2}}\frac{\exp(-\frac{1}{2}{\bar{u}}^{T}{\left(W^{T}W\right)}^{-1}\bar{u})}{2\pi \sqrt{\det(W^{T}W)}}\;d\bar{u}.$$

In other words, $\Phi_{2}\left(z_{1},z_{2},z_{3}\right)$ denotes the multivariate integral in two standard normal variables with a correlation coefficient of $z_{3}$, evaluated with limits as seen in Equation (30). Finally, we use the multivariate PDF for $\bar{z}$:

(31) $$E\left[T_{a}^{2}\;|\;{\bar{m}}_{a}\right]=\int_{R^{2}\times \left(-1,1\right)}\frac{\exp(-\frac{1}{2}{\left(\bar{z}-{\bar{\mu }}_{2}\right)}^{T}\Sigma_{2}^{-1}\left(\bar{z}-{\bar{\mu }}_{2}\right))}{\sqrt{{\left(2\pi \right)}^{3}\det\left(\Sigma_{2}\right)}}\Phi_{2}{\left(z_{1},z_{2},z_{3}\right)}^{A-1}d\bar{z},$$

with ${\bar{\mu }}_{2},\Sigma_{2}$ presented above in (28).

### 4.5. Conditioned Market Share Expectation

In this section, we derive estimates for the expected market share of alternative *a* in a random market, when the alternative is conditioned in a way that gives us some partial information about its measurement vector.

For the rest of the section, we will be working with normalized weights and applying Theorem A3 to obtain ${\bar{\omega }}_{i}\cdot {\bar{m}}_{a}\;|\;{\bar{\omega }}_{i}∼N\left(0,{\left\|{\bar{\omega }}_{i}\right\|}^{2}\right)=N\left(0,1\right)$. Note that, for $a\neq a^{\prime }$, we have $\mathrm{cov}({\bar{\omega }}_{i}\cdot {\bar{m}}_{a},\;{\bar{\omega }}_{i}\cdot {\bar{m}}_{a^{\prime }}\;|\;{\bar{\omega }}_{i})=0$. This implies that we can treat $v_{i,a}$ and each $v_{i,a^{\prime }}$ as independent normal variables inside the integral. We begin with a simple sanity check by deriving the mean market share $E_{M}\left[T_{a}\right]$, which has to equal 1/A due to symmetry:

(32) $$E_{M}\left[T_{a}\right]=\int_{\Omega_{W}}P_{M}\left(v_{i,a}>v_{i,a^{\prime }},\;\forall a^{\prime }\neq a\;|\;{\bar{\omega }}_{i}\right)\;dP\left({\bar{\omega }}_{i}\right)\approx \int_{-\infty }^{\infty }\Phi {\left(x\right)}^{A-1}\phi \left(x\right)\;dx=\frac{1}{A}.$$

Our strategy henceforth will be to impose some restriction on ${\bar{m}}_{a}$ in the form of it belonging to an event *R* that has a non-zero measure, such that, given ${\bar{\omega }}_{i}$ and *R*, $v_{i,a}$ is normally distributed. Since ${\mathrm{cov}}_{M}\left({\bar{\omega }}_{i}\cdot {\bar{m}}_{a},\;{\bar{\omega }}_{i}\cdot {\bar{m}}_{a^{\prime }}\;|\;{\bar{\omega }}_{i},\;R\right)=0$, we can solve for the market share expectation $E_{M_{a}}\left[T_{a}\;|\;{\bar{m}}_{a}\in R\right]$ as we did in Section 4.3. As in Theorem A4, some requirements on the moments are needed to ensure uniform convergence over $\Omega_{W}$.

#### 4.5.1. Conditioning on the Norm of the Measurement Vector

Here, we investigate what we can tell about the market share of an alternative, knowing only the norm of its measurement vector. If the alternative stands out in some way, even one that is not universally appreciated, how would that affect its market share? Assume that in our market, the measurements are symmetric in the sense that $E[{\left(m_{a}^{f}\right)}^{3}]=0$, and that we have an alternative ${\bar{m}}_{a}$ with $\|{\bar{m}}_{a}\|=r\sqrt{F}$ for some r>0.

Consider ${\bar{\omega }}_{i}\cdot {\bar{m}}_{a}\;|\;{\bar{\omega }}_{i}∼N\left(0,1\right)$ and ${\left\|{\bar{m}}_{a}\right\|}^{2}∼N\left(F,\sum_{f=1}^{F}E[{\left(m_{a}^{f}\right)}^{4}]-1\right)$ as bivariate normal random variables. Their correlation is

(33) $$\mathrm{cor}\left({\bar{\omega }}_{i}\cdot {\bar{m}}_{a}\;|\;{\bar{\omega }}_{i},{\left\|{\bar{m}}_{a}\right\|}^{2}\right)=\frac{\sum_{f=1}^{F}\omega_{i}^{f}E\left[{\left(m_{a}^{f}\right)}^{3}\right]}{\sqrt{\sum_{f=1}^{F}E[{\left(m_{a}^{f}\right)}^{4}]-1}}.$$

This is a random variable which is dependent on ${\bar{\omega }}_{i}$. But since we are working with a symmetric market, it reduces to zero. Using the equations for bivariate normal variable conditioning,

(34) $${\bar{\omega }}_{i}\cdot {\bar{m}}_{a}\;|\;{\bar{\omega }}_{i},\|{\bar{m}}_{a}\|=r\sqrt{F}∼N\left(0,1\right).$$

At very large values of *F*, this limit does indeed hold, but a better approximation can be made using the law of large numbers (LLN) (in order to allow for the law of large numbers with our non identically generated $E[{\left(m_{a}^{f}\right)}^{3}]$, we either need to assume additional mild conditions on the model generation (for example, independence of $E[{\left(\omega_{i}^{f}\right)}^{2}]$ and $E[{\left(m_{a}^{f}\right)}^{3}]$), or use some special version of the LLN) to show that

(35) $$\mathrm{var}\left({\bar{\omega }}_{i}\cdot {\bar{m}}_{a}\;|\;{\bar{\omega }}_{i},\|{\bar{m}}_{a}\|=r\sqrt{F}\right)=\sum_{f=1}^{F}{\left(\omega_{i}^{f}\right)}^{2}E\left[{\left(m_{a}^{f}\right)}^{2}\;|\;\|{\bar{m}}_{a}\|=r\sqrt{F}\right]\underset{F\to \infty }{\to }r^{2}.$$

Using ${\bar{\omega }}_{i}\cdot {\bar{m}}_{a}∼N\left(0,r^{2}\right)$ we have

(36) $$E_{M}\left[T_{a}\;|\;\|{\bar{m}}_{a}\|=r\sqrt{F}\right]\approx \frac{1}{r}\int_{-\infty }^{\infty }\Phi {\left(x\right)}^{A-1}\phi \left(\frac{x}{r}\right)\;dx.$$

Or, alternatively,

(37) $$E_{M}\left[T_{a}\;|\;\|{\bar{m}}_{a}\|=r\right]\approx \frac{\sqrt{F}}{r}\int_{-\infty }^{\infty }\Phi {\left(x\right)}^{A-1}\phi \left(\frac{\sqrt{F}x}{r}\right)\;dx.$$

**Figure 6:**
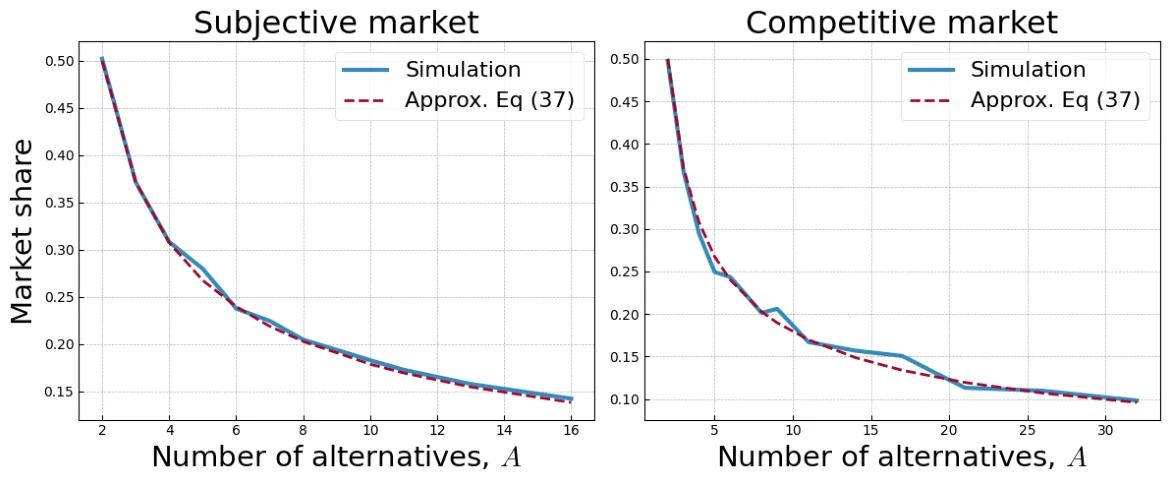
A comparison of simulated data and the general estimate for the conditioned norm expected value $E_{M}\left[T_{a}\;|\;\|{\bar{m}}_{a}\|=\sqrt{F}+1\right]$ using normal distributions for weights and measurements (which is a symmetric distribution) and F=30.

Figure 6 demonstrates that the approximation is accurate when using a normal distribution for the measurements due to its zero skew.

#### 4.5.2. Conditioning on the Value an Individual Assigns to an Alternative

We now consider another case where partial information is known about an alternative. Specifically, assume we have a single individual evaluate the information based on their preference (weight vector, denoted as $\bar{u}$), which is fully known. For simplicity, we take $\|\bar{u}\|=1$. We are interested in the conditional expectation of the market share of alternative *a* given $\bar{u}\cdot {\bar{m}}_{a}=v$, $E_{M}\left[T_{a}\;|\;\bar{u}\cdot {\bar{m}}_{a}=v\right]$. The idea here is that we can split the product ${\bar{\omega }}_{i}\cdot {\bar{m}}_{a}$ into the linear projection and rejection (which is the perpendicular component) of vector ${\bar{\omega }}_{i}$ onto $\bar{u}$:

(38) $$E_{M}\left[T_{a}\;|\;\bar{u}\cdot {\bar{m}}_{a}=v\right]=\int_{\Omega_{W}}P\left(\left({\mathrm{proj}}_{\;\bar{u}}{\bar{\omega }}_{i}+{\mathrm{oproj}}_{\;\bar{u}}{\bar{\omega }}_{i}\right)\cdot {\bar{m}}_{a}>{\bar{\omega }}_{i}\cdot {\bar{m}}_{a^{\prime }}\;\forall a^{\prime }\neq a\;|\;{\bar{\omega }}_{i}\right)\;dP\left({\bar{\omega }}_{i}\right),$$

where

(39) $${\mathrm{proj}}_{\;\bar{u}}{\bar{\omega }}_{i}=\frac{\bar{u}\cdot {\bar{\omega }}_{i}}{{\left\|\bar{u}\right\|}^{2}}\bar{u}=\left(\bar{u}\cdot {\bar{\omega }}_{i}\right)\bar{u},\;{\mathrm{oproj}}_{\;\bar{u}}{\bar{\omega }}_{i}={\bar{\omega }}_{i}-{\mathrm{proj}}_{\;\bar{u}}{\bar{\omega }}_{i}={\bar{\omega }}_{i}-\left(\bar{u}\cdot {\bar{\omega }}_{i}\right)\bar{u}.$$

Now, since the vectors are orthogonal, we write $\left\|{\mathrm{oproj}}_{\;\bar{u}}{\bar{\omega }}_{i}\right\|=\sqrt{1-{\left(\bar{u}\cdot {\bar{\omega }}_{i}\right)}^{2}}$ and notice that

(40) $$\begin{array}{cc} \mathrm{cov}({\mathrm{proj}}_{\;\bar{u}}{\bar{\omega }}_{i}\cdot {\bar{m}}_{a},\;{\mathrm{oproj}}_{\;\bar{u}}{\bar{\omega }}_{i}\cdot {\bar{m}}_{a}\;|\;{\bar{\omega }}_{i}) & =E\left[\sum_{f=1}^{F}{m_{a}^{f}}^{2}{\left({\mathrm{proj}}_{\;\bar{u}}{\bar{\omega }}_{i}\right)}_{f}{\left({\mathrm{oproj}}_{\;\bar{u}}{\bar{\omega }}_{i}\right)}_{f}\;|\;{\bar{\omega }}_{i}\right]\\ & ={\mathrm{proj}}_{\;\bar{u}}{\bar{\omega }}_{i}\cdot {\mathrm{oproj}}_{\;\bar{u}}{\bar{\omega }}_{i}=0. \end{array}$$

Hence, they are jointly normal. We can, therefore, write

(41) $$\left({\bar{\omega }}_{i}\cdot {\bar{m}}_{a}\;|\;{\bar{\omega }}_{i},\bar{u}\cdot {\bar{m}}_{a}=v\right)∼N\left(\left(\bar{u}\cdot {\bar{\omega }}_{i}\right)v,\;1-{\left(\bar{u}\cdot {\bar{\omega }}_{i}\right)}^{2}\right).$$

In this case, the means and variances of the value distribution are dependent but still normal for a given weight vector. We can solve this by going directly to the integral (38):

(42) $$\begin{array}{cc} \; & \int_{\Omega_{W}}P\left(\left(\bar{u}\cdot {\bar{\omega }}_{i}\right)v+\sqrt{1-{\left(\bar{u}\cdot {\bar{\omega }}_{i}\right)}^{2}}Z_{a}>Z_{a^{\prime }}\;\forall a^{\prime }\neq a\right)\;dP\left({\bar{\omega }}_{i}\right)\\ \; & =\int_{\Omega_{W}}\frac{1}{\sqrt{1-{\left(\bar{u}\cdot {\bar{\omega }}_{i}\right)}^{2}}}\int_{-\infty }^{\infty }\Phi {\left(x\right)}^{A-1}\phi \left(\frac{x-(\bar{u}\cdot {\bar{\omega }}_{i})v}{\sqrt{1-{\left(\bar{u}\cdot {\bar{\omega }}_{i}\right)}^{2}}}\right)\;dx\;dP\left({\bar{\omega }}_{i}\right). \end{array}$$

There are two specific cases we may be interested in:

1. The ‘information’ vector has a shape that will lead to $\bar{u}\cdot {\bar{\omega }}_{i}$ being distributed normally. This happens as long as the magnitude of the weights is not concentrated too much on a small subset of features.
2. The case where $\bar{u}=e_{f}$, a standard basis vector, which allows us to check the effect of an isolated feature and its measurement on the alternative’s market share.

In the first case, $\bar{u}\cdot {\bar{\omega }}_{i}∼N\left(\bar{u}\cdot {\bar{\mu }}_{W},\;{\bar{u}}^{2}\cdot {\bar{\sigma }}_{W}^{2}\right)$. Therefore,

(43) $$\int_{-\infty }^{\infty }\int_{-1}^{1}\frac{1}{\sqrt{\left(1-z^{2}\right)\left({\bar{\sigma }}_{W}^{2}\cdot {\bar{u}}^{2}\right))}}\Phi {\left(x\right)}^{A-1}\phi \left(\frac{x-vz}{\sqrt{1-z^{2}}}\right)\phi \left(\frac{z-{\bar{\mu }}_{W}\cdot \bar{u}}{\sqrt{{\bar{\sigma }}_{W}^{2}\cdot {\bar{u}}^{2}}}\right)\;dz\;dx.$$

In the second case, we are going to need the distribution density function for $\omega_{i}^{f}$, denoted as $\varphi_{f}\left(z\right)$:

(44) $$\int_{-\infty }^{\infty }\int_{-1}^{1}\frac{1}{\sqrt{1-z^{2}}}\Phi {\left(x\right)}^{A-1}\phi \left(\frac{x-vz}{\sqrt{1-z^{2}}}\right)\varphi_{f}\left(z\right)\;dz\;dx.$$

**Figure 7:**
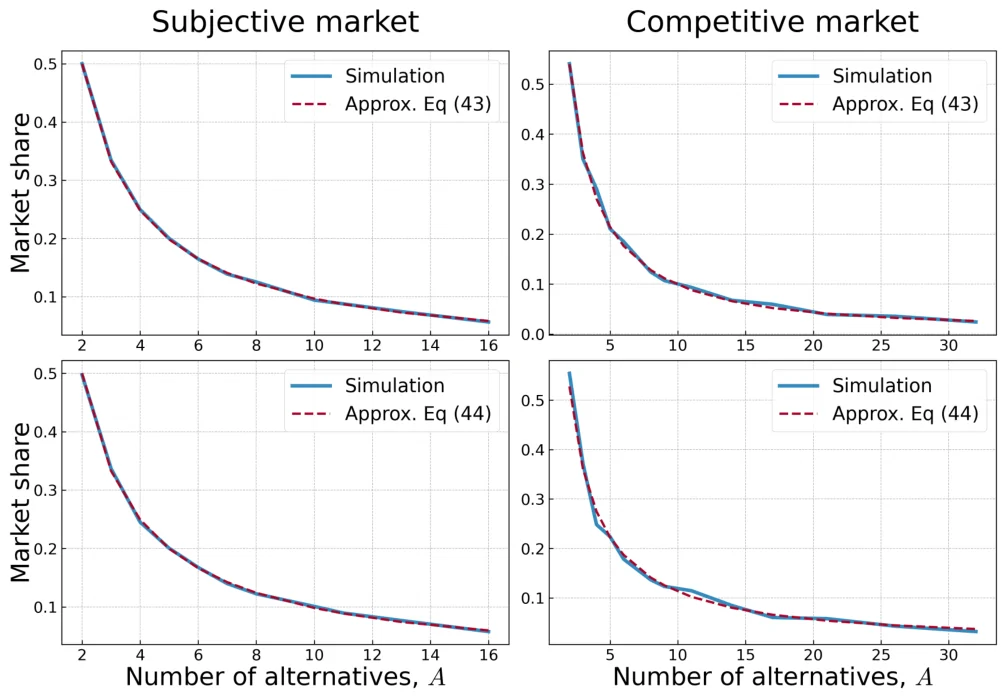
A comparison of simulated data and the estimate for the expected value of an alternative by conditioning on its subjective value for some test weight vector $\bar{u}$. (**Top**): $\bar{u}$ was picked as a random Gaussian vector. (**Bottom**): $\bar{u}=e_{f}$, which is akin to singling out a measurement $m_{a}^{f}$ and judging based on it alone. In both cases, F=30.

Figure 7 shows a comparison between simulations and approximations.

### 4.6. Distribution of Market Share

In this section, we derive a formula for the comparison distribution, which is the market share distribution of size A=2, and use it to calculate the comparison entropy. We also provide ad hoc bounds for the market share distribution of competitive markets.

Using the Gaussian approximation, finding a formula for $P(T_{a}<t)$ for A=2 is relatively simple since there are only two alternatives to compare:

(45) $$T_{a}\left({\bar{m}}_{a},{\bar{m}}_{a^{\prime }}\right)=P\left(v_{i,a}>v_{i,a^{\prime }}\;|\;{\bar{m}}_{a},{\bar{m}}_{a^{\prime }}\right)=\Phi \left(\frac{{\bar{\mu }}_{W}\cdot \left({\bar{m}}_{a}-{\bar{m}}_{a^{\prime }}\right)}{\sqrt{{\bar{\sigma }}_{W}^{2}\cdot {\left({\bar{m}}_{a}-{\bar{m}}_{a^{\prime }}\right)}^{2}}}\right).$$

Then,

(46) $$P\left(T_{a}<t\right)=P\left(\frac{{\bar{\mu }}_{W}\cdot \left({\bar{m}}_{a}-{\bar{m}}_{a^{\prime }}\right)}{\sqrt{{\bar{\sigma }}_{W}^{2}\cdot {\left({\bar{m}}_{a}-{\bar{m}}_{a^{\prime }}\right)}^{2}}}<\Phi^{-1}\left(t\right)\right)=\Phi \left(\eta \;\Phi^{-1}\left(t\right)\right).$$

**Figure 8:**
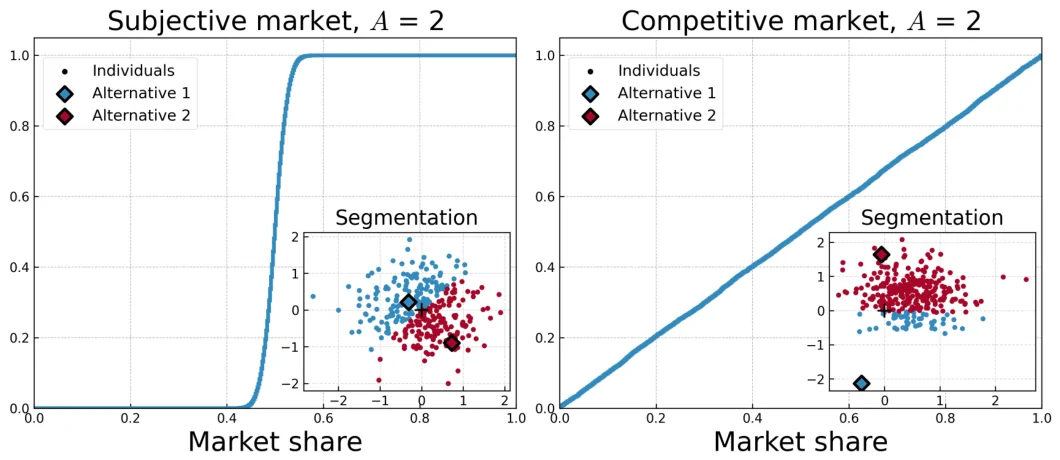
CDFs of markets of size two. Insets show the segmentation of the market by chosen alternatives, where both alternatives and their respective segments are colored accordingly. Small dots represent individuals’ weight vectors, colored by their preferred alternative. Diamonds represent the alternatives. The boundary of each segment must go through the center of axes, which is marked with a plus. In a subjective market, two alternatives always segment the market into two parts going through the origin. As F→∞, the CDF of the subjective market distribution approaches a step function. In a competitive market with two alternatives, as F→∞, the distribution of market shares becomes uniform.

We can see that when the market is subjective, as in $\eta =\|{\bar{\sigma }}_{W}\|/\|{\bar{\mu }}_{W}\|\to \infty$, the inner term will be close to −∞ when t<1/2 and +∞ when t>1/2. This implies that as F→∞, the CDF approaches a step function at 1/2. When η=1, Equation (46) simplifies into $P(T_{a}<t)=t$, a uniform distribution, as can be seen in Figure 8.

The fact that a market has a uniform distribution when comparing two alternatives is significant, as it implies that the competitive distribution maximizes the comparison entropy, which is how we originally defined the competitive distribution. In other words, a competitive market is one in which any two random alternatives segment the market in the least predictable way. From this fact, we draw the justification for calling it a competitive market.

Recall our definition of comparison entropy back in Section 2.2. In the context of the linear preference model, using Equation (46),

(47) $$c_{\eta }\left(x\right)=\frac{\eta \phi \left(\eta \Phi^{-1}\left(x\right)\right)}{\phi (\Phi^{-1}\left(x\right))},$$

(48) $$\begin{array}{cc} H[(W,M)] & =-\int_{0}^{1}\frac{\eta \phi \left(\eta \Phi^{-1}\left(x\right)\right)}{\phi (\Phi^{-1}\left(x\right))}\log\left(\frac{\eta \phi \left(\eta \Phi^{-1}\left(x\right)\right)}{\phi (\Phi^{-1}\left(x\right))}\right)dx. \end{array}$$

Substituting $z=\Phi^{-1}\left(x\right)$,

(49) $$\begin{array}{cc} H[(W,M)] & =-\int_{-\infty }^{\infty }\eta \phi \left(\eta z\right)\left(\log\left(\eta \right)+\log\left(\phi \left(\eta z\right)\right)-\log\left(\phi \left(z\right)\right)\right)dz\\ \; & =-\log\left(\eta \right)+\frac{\eta^{2}-1}{2\eta^{2}}. \end{array}$$

Maximizing (49), we find that the maximal entropy distribution is obtained for η=1. Accordingly, this is our definition of a competitive market. This also follows on directly from the fact that the maximal entropy distribution among those defined on [0,1] is uniform when η=1.

Based on Equation (47), we can also derive a maximum-likelihood estimator for η. Given *n* pairwise comparisons where $0<t_{i}<1$ of the population choose one of the alternatives, one can easily find the MLE to be

(50) $$\hat{\eta }=\sqrt{\frac{n}{\sum_{i=1}^{n}\Phi^{-1}{\left(t_{i}\right)}^{2}}}.$$

**Figure 9:**
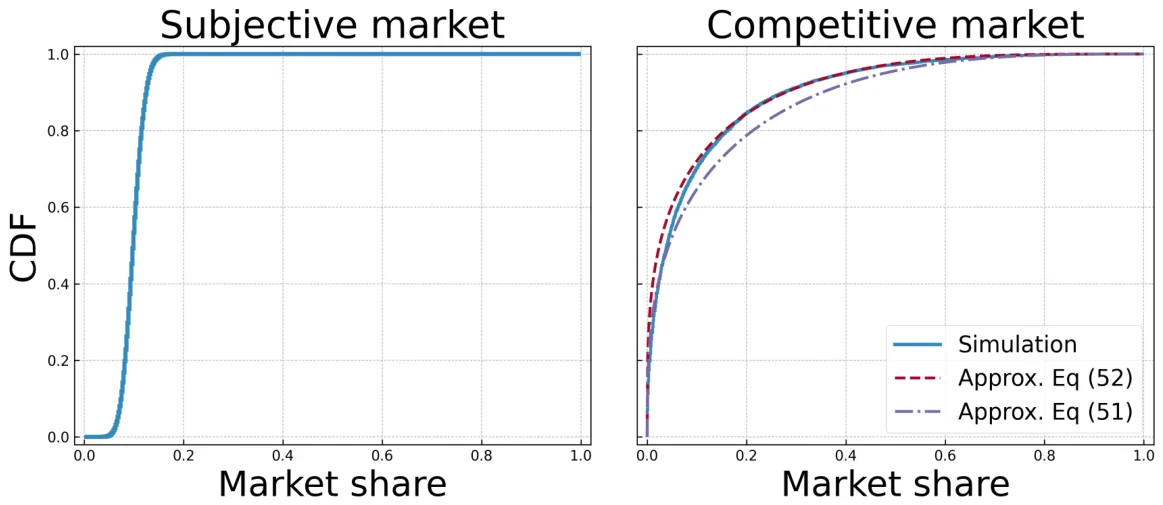
Empirical cumulative distribution function of both subjective and competitive markets with A=10. For the competitive distribution, we provide two rough approximations. The true simulated value appears to be in between those two curves.

Using the same simulation as above, we investigate the market share distribution for larger values of *A* (Figure 9). The competitive distribution appears to be similar in shape to an $L^{p}$ circle with p=A−1. This would provide a distribution of

(51) $$P\left(T_{a}<t\right)=(1-{\left(1-t\right)}^{{\left(A-1\right)}^{\frac{1}{2}}}{)}^{{\left(A-1\right)}^{-\frac{1}{2}}}.$$

In fact, this approximation appears to work quite well as a lower bound (Figure 9), together with an upper bound provided by approximately

(52) $$P\left(T_{a}<t\right)={\left(1-{\left(1-t\right)}^{{\left(A-1\right)}^{\gamma }}\right)}^{{\left(A-1\right)}^{-\gamma }},$$

where γ≈0.5772 is the Euler–Mascheroni constant.

## 5. Imperfect Information and Softmax Choice

We now turn our attention to markets with incomplete information. We can model such markets by employing the softmax decision rule, instead of having individuals pick their best alternative with probability 1.

As shown in reference [14], multinomial logit choice probabilities arise from a random utility model in which each alternative is provided by $U_{i,a}=v_{i,a}+\epsilon_{i,a}$, where the $\epsilon_{i,a}$ are i.i.d. type I extreme value (Gumbel) random variables. We can work that into our case by introducing choice probabilities:

(53) $$P_{i}\left(a\right)=\frac{e^{\beta v_{i,a}}}{\sum_{a^{\prime }=1}^{A}e^{\beta v_{i,a^{\prime }}}},$$

where 0<β<∞ is the inverse-temperatureparameter, which resembles the amount of information available in the market. In the limit β→∞, we obtain our original best-choice decision rule. As seen in reference [33], the noise term $\epsilon_{i,a}$ follows a Gumbel distribution with zero location and scale 1/β. We can roughly approximate it with a normal distribution of the same variance, center it (the mean of the noise does not affect choice probabilities), and use it to approximate $\eta_{\mathrm{eff}}$ as a function of β:

$$v_{i,a}+\epsilon_{i,a}∼N\left(0,1+\frac{\pi^{2}}{6\beta^{2}}\right),$$

(54) $$\eta_{\mathrm{eff}}=\frac{\sqrt{{\left\|{\bar{\sigma }}_{W}\right\|}^{2}+\frac{\pi^{2}}{6\beta^{2}}}}{\left\|{\bar{\mu }}_{W}\right\|}=\sqrt{\eta^{2}+\frac{\pi^{2}\left(1+\eta^{2}\right)}{6\beta^{2}}}.$$

**Figure 10:**
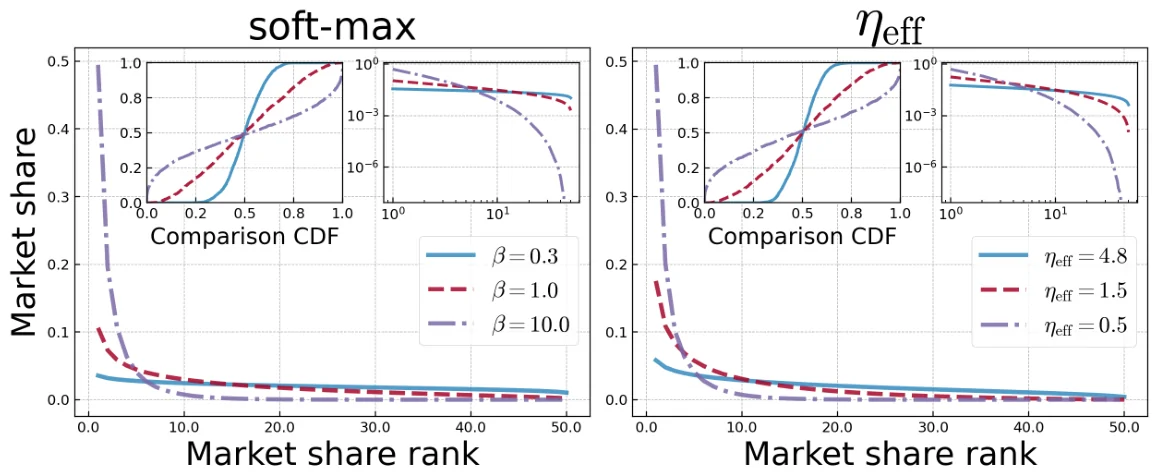
(**Left**) Rank frequency plots generated using the softmax choice rule with η=0.5 and a varying inverse-temperature β. (**Right**) Rank frequency plots averaged over many market realizations using the best value choice rule with the appropriate $\eta_{\mathrm{eff}}$. Small inconsistencies may arise due to approximating the Gumbel noise by the normal distribution. (**Left inset**): Comparison distribution CDF in each case. (**Right inset**): Log–log plot of the rank frequency plots.

Figure 10 shows the rank frequencies and comparison distributions in markets using the softmax choice rule, as well as the same plots for markets using the best choice rule with a modified $\eta_{\mathrm{eff}}$. The market structure itself stays mostly intact, suggesting that adding noise to the market, for example, by means of faulty information, or lack thereof, can masquerade as an inherent higher degree of subjectivity (for discussion on similar ideas, see [34]).

This result suggests an underlying connection between systematic utility distributions generated by a linear preference model and the random utility theory framework presented in [12,15] and other works.

In Section 6, we discuss the model’s connection to neural networks and provide a short example of a preference market occurring in classification data in a natural manner. In Appendix C and Appendix D, we introduce market segmentation and present initial results in deriving a general formula for the market share distribution.

## 6. Relation to Classification Neural Networks

We present an example of a preference market arising naturally from a classification neural network by likening classification probabilities to utility. The purpose of this experiment is to compare theory with data and test the model predictions, specifically the importance of the parameter η with non-linear utilities.

### 6.1. Problem Setup

**Figure 11:**
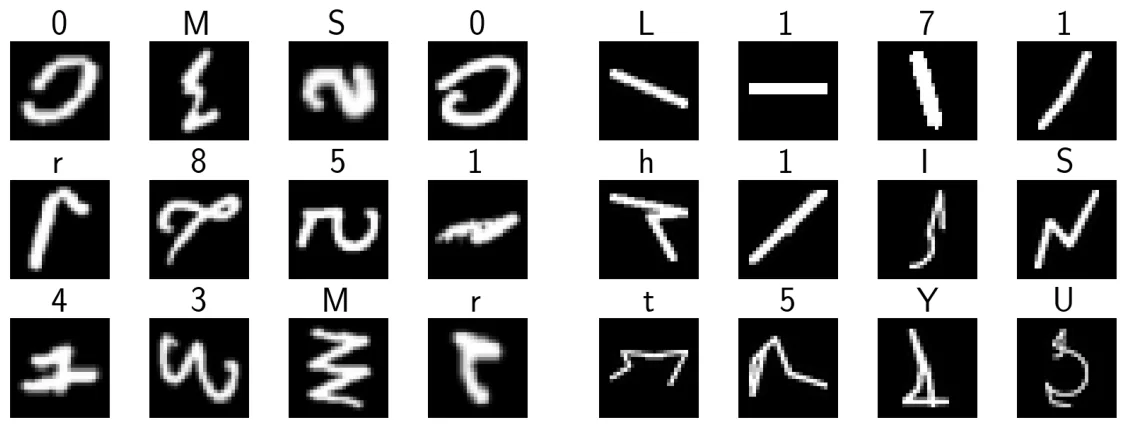
(**Left**) Example characters from the MNIST extended dataset. Above each character, the digit/letter that the classifier assigns to it is displayed. (**Right**) Randomly generated symbols from three distinct sets with varying amounts of lines. (**Top**) Few lines. (**Center**) Medium lines. (**Bottom**) Many lines.

First, a neural network with multiple hidden layers was trained (the network received an input of a 28 × 28 grayscale image, which was processed through two convolutional blocks, followed by a flat dense layer and then a softmax layer with 47 elements for the various characters. It uses ReLU activations, dropout for regularization, and batch normalization to stabilize training) to classify images into English letters and digits; henceforth, characters using the extended MNIST dataset [35]. We then generated synthetic symbols using a custom Python (https://www.python.org/) script that draws random scribbles with varying structural complexity (the symbols were drawn using a turtle script [36] with two basic operations: draw a line in a random direction or draw a circle arc in a random direction, starting from the last position in both cases. Angles and lengths were picked out of configurable distributions, alongside probabilities to draw a straight line or an arc. The number of lines drawn was the only parameter to be changed between the three data sets, ranging from 1–2 in the “few lines” set to 5–7 in the “many lines” set), henceforth, symbols. These synthetic symbols were passed through the trained classifier to determine which label, or character, they most closely resembled. Figure 11 shows some of the characters and symbols, along with their classifications by the neural network.

One can think of a decision process as a machine that takes an input describing an individual (weights) and uses them to pick one of the alternatives [28]. In the classification neural networks studied here, characters play the role of alternatives and symbols as weights. The relevant features and measurements for each alternative are found by training the network. The process of classification corresponds to choosing the most appropriate character. In fact, if the classification network were to be composed of only the first and last layers, it would result in the match probability being a monotone transformation (typically softmax) of a linear product, resulting in the same preference order as that of a linear utility.

**Figure 12:**
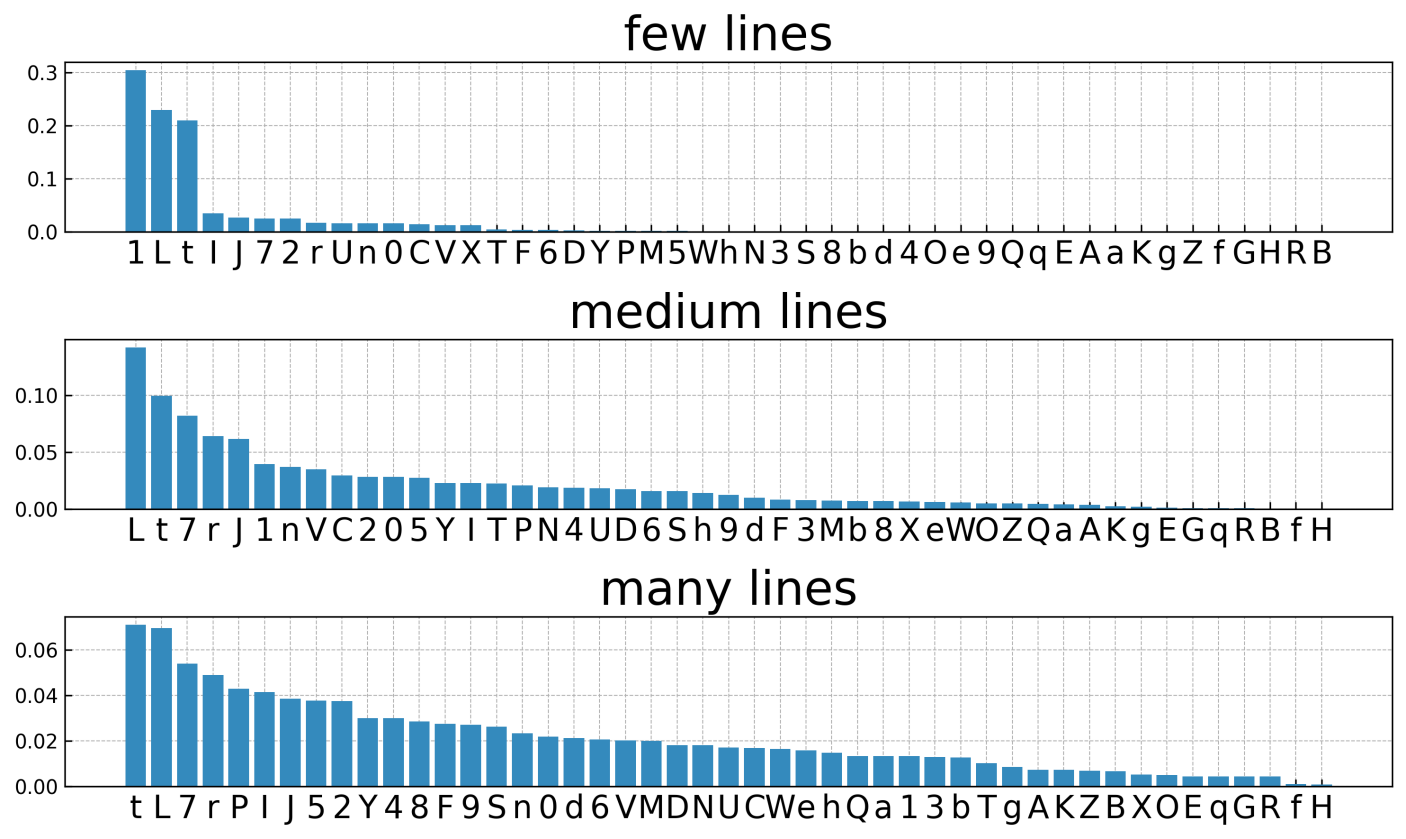
Rank-frequency plots of the random symbols generated in each set. Each set was generated with the same configurations, aside from the number of lines drawn. The number of lines drawn ranges from 1–2 in the “few lines” set and 5–7 in the “many lines” set.

Figure 12 shows the rank-frequency plots of random symbols in each set. Intuitively, different symbols with many lines are often interpreted as different characters, which is analogous to a subjective market. In contrast, with a smaller number of lines, most symbols are interpreted as ’1’, ’L’, or ’t’, leading to a more objective market.

### 6.2. Comparison Distribution

**Figure 13:**
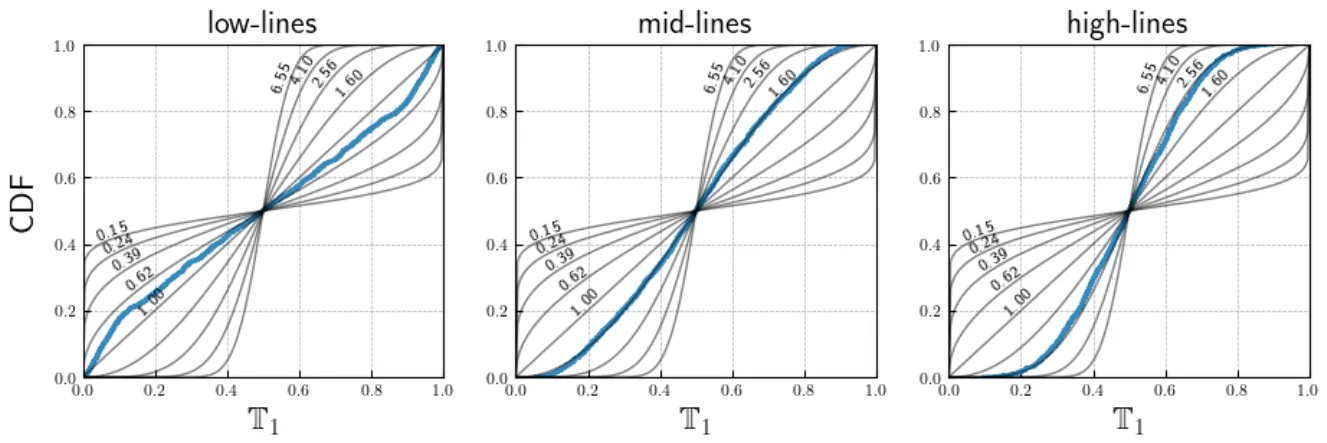
Empirical comparison distribution for each symbol set in blue. In black, in the background, we have displayed a sample of some set values of η using Equation (46). The empirical comparison distribution was generated by creating a histogram from all possible pairs of alternatives (characters). For any given pair, we note the percentage of the population (symbols) that would pick one alternative over the other. We do this by comparing the respective values in the final vector of the neural network, i.e., the output layer, for each symbol. This gives rise to a distribution with symmetry around 1/2, as the order of comparison does not matter.

Because the final layer of a classification network is a full vector of probabilities, we can compare any two characters and derive the empirical comparison distribution for the market (Figure 13). By minimizing the Kolmogorov–Smirnov statistic on the comparison distribution, we can estimate values of η as 0.81, 1.61, and 2.32 for the respective data sets. Note that these estimates are only empirical, since the model is defined for linear utility, which digit classification is unlikely to represent. Still, not only do we see that the empirical comparison distribution is shaped mostly as we expect it to be in a linear preference market, but we can also observe how a larger number of lines in the symbol set leads to a higher value of η, as in a larger degree of subjectivity in the market.

In Section 7.3, we present an argument for why non-linear utility might still adhere to the shape of a linear preference model more often than expected.

## 7. Discussion

In this paper, we studied a high-dimensional probabilistic formulation of the linear multi-attribute utility model and investigated the statistical behavior of preference markets arising from it. While traditional approaches to MCDA typically emphasize the structure of individual utility functions and discrete choice modeling, our perspective shifts toward population-level outcomes.

To characterize the impact of variation in individual preferences, we introduced the parameter $\eta =\|{\bar{\sigma }}_{W}\|/\|{\bar{\mu }}_{W}\|$, the degree of subjectivity in a market. Markets with η approaching zero exhibit largely objective behavior, where most individuals rank alternatives similarly. In contrast, when η is large, markets become highly subjective, and choice outcomes depend strongly on individual differences. Of particular interest is the case η=1, which defines a competitive market. There, the comparison distribution becomes uniform, indicating maximal entropy and minimal predictability: an emergent balance between consensus and variation.

We then derived analytical approximations for the expected market share of a fixed alternative in a random market, as well as for its variance and several forms of conditional expectations. These results support our main observation that the single parameter η classifies the distribution of market share among the alternatives.

Finally, we demonstrated the model’s applicability through a simulation involving randomly generated symbols and a trained neural network classifier. In this experiment, the neural network assigned labels to synthetic inputs, and the resulting distribution of classification outcomes naturally mirrored those of preference markets under the linear model. The similarity between empirical and simulated distributions suggests that the framework captures relevant features of high-dimensional decision processes and points to its potential use as a diagnostic tool in practical domains such as recommender systems, polling, and behavioral sciences.

### 7.1. Context and Repeated Surveying

Context is an important factor for preference. For example, asking individuals to pick their favorite fruit out of a list, which they may not have experienced in its entirety, could produce different results than first providing them with samples. In order to produce consistent and meaningful results when analyzing a number of decision-making processes, it is important that the context is consistent. This consistency is paramount for conducting a proper statistical analysis of preference, and while we took it for granted when defining the model, it is important to remember it is not trivial when working with real data and polls.

When the decision problem is consistent, each individual is met with the exact same scenario, and differences in individual preference alone cause differences in choice. The context can be thought of as an anchor, as discussed in [37].

An interesting idea is to transfer the implications of a change in context into a change in the individual’s preference. In theory, a probability distribution of contexts could then be transformed into a distribution of preference, the same as is possessed by the entire population (albeit being different parametrically).

One realization of this idea can be formed by conducting repeated surveys over time for a single individual, which may, in turn, approximate a cross-sectional survey under ergodicity assumptions.

### 7.2. Practical Limitations of the Model

#### 7.2.1. Inherent Limitations of the Linear Preference Model

The most apparent limitation of modeling utility linearly is the inability to account for features that enhance or detract from one another. A fixed non-linear transformation of a single feature can be handled by transforming that measurement and its distribution (see Appendix E), but this does not account for interactions between features or individual-specific transformations (A simple example can be found in cooking: two ingredients may taste good separately, but not when mixed together). In Section 7.3, we provide an informal argument for how some non-linear utility functions may be represented using linear utility at the cost of introducing cross-weight correlations. Section 6 also presents one potentially non-linear case in which the empirical comparison distribution remained close to that predicted by the linear model. These observations suggest that the framework may remain useful in some non-linear settings, but we do not prove robustness to non-linear interactions or cross-weight correlations in this paper.

Aside from linearity, the model assumes complete and common information about the alternatives, decisions made independently by each individual, and an unlimited supply. Prices may be included as fixed measurements, but endogenous pricing, capacity constraints, and strategic or social interactions between decision-makers are not modeled. Section 5 relaxes deterministic choice by introducing random-utility noise through the softmax rule.

The model also describes a static market with a consistent context. In real markets, measurements, preferences, available alternatives, and consideration sets may change over time, while previous choices may themselves affect later decisions. Pooling observations across such changes can violate the assumed stability and independence of the underlying distributions. Context effects introduce a related limitation, discussed in Section 7.1: apparent differences in preference may instead reflect differences in how the same decision problem is presented or interpreted.

#### 7.2.2. Limitations Arising from High-Dimensional Limit Assumptions

When deriving the equations for the expected market share in Theorem A3 (Appendix B), we had to take the assumption that

$$\sum_{f=1}^{F}E\left[{\left(\omega_{i}^{f}\right)}^{4}\right]\underset{F\to \infty }{⟶}0,$$

as well as

$$\sum_{f=1}^{F}E[|\omega_{i}^{f}{|}^{3}]\cdot E[|m_{a^{\prime }}^{f}{|}^{3}]\underset{F\to \infty }{\to }0.$$

One of the major drawbacks of these conditions is that it becomes challenging to include distributions that are not sub-Gaussian.

The first condition essentially comes down to requiring that the magnitude of the weights is spread apart throughout the features and not clumped up too much in a small number of over-dominant features. It is also connected to the participation ratio of ${\bar{\sigma }}_{W}^{2}$, defined as ${\left\|{\bar{\sigma }}_{W}\right\|}_{2}^{4}/{\left\|{\bar{\sigma }}_{W}\right\|}_{4}^{4}$ in the case the vector’s elements are independent, and also sometimes referred to as the effective dimensionality of the vector. Indeed, since $E\left[{\left(\omega_{i}^{f}\right)}^{4}\right]\geq {\left(\sigma_{W}^{f}\right)}^{4}$, the first condition forces this effective dimension to diverge whenever the total variance stays bounded away from zero; the converse additionally requires control of the fourth moments. In some scenarios, we might find that while *F* itself is large, the effective dimensionality is the real limiter to the model’s applicability.

The second condition can be thought of as related to deal breakers. In order to fail, the condition would require some features whose weights and/or measurements are highly skewed.

For example, take food allergies as a deal breaker for an individual choosing a meal from a menu in a restaurant. For some individuals, the weight of the deal breaker or allergen will be extraordinarily large, which will invalidate, in a sense, the usual form of decision making.

Additionally, when measuring the expected market share for some given alternative with known measurements ${\bar{m}}_{a}$, we had to further assume that

$$\underset{F}{\sup}\left|\frac{{\bar{m}}_{a}\cdot {\bar{\mu }}_{W}\left(F\right)}{\sqrt{{\bar{m}}_{a}^{2}\left(F\right)\cdot {\bar{\sigma }}_{W}^{2}\left(F\right)}}\right|<\infty$$

on our limit of F→∞. The implication of this is that the expected value estimate starts failing for *superstar* alternatives—if the value of the alternative is sufficiently above the typical range for the other alternatives, the approximation might fail. This could become relevant in regimes where the size of the market *A* is comparable to, or grows with, the size of the population *I*. In such regimes, such as the population of cities, the top alternatives’ values could approach such limits due to the laws of extreme-value theory. The error itself would vanish as *F* grows, but it raises a question about what if *F* itself grows as a function of *I*. Such regimes with many (with respect to *I*) alternatives are an interesting topic in and of themselves, but they remain outside the scope of this paper.

#### 7.2.3. Limitations of Empirical Interpretation

Linear additivity, independence, moment conditions, and a sufficiently large effective dimension are mathematical limitations of the present theory, since they are used to derive the limiting market-share laws. Other limitations primarily affect empirical interpretation. Within the model, η measures preference heterogeneity relative to the shared component of preference. In observed choices, however, similar dispersion may arise from decision noise, incomplete information, limited consideration, changing context, or resource-limited information processing. Aggregate market shares alone do not generally distinguish between these mechanisms. An empirically fitted value of η should, therefore, be interpreted as a model-conditional description of the observed comparison distribution, rather than as identifying preference heterogeneity as its unique cause. Separating these explanations would require additional structure or data, such as repeated choices, controlled variation in menus or information, or direct measurements of the decision process.

### 7.3. Non-Linearity and Cross-Weight Correlations

Looking back at Figure 13, one might suspect there is more going on behind the scenes to allow for a non-linear problem to so closely align with a linear model. We provide a non-rigorous argument for how one can attempt to model any analytic utility function using a linear model, assuming that correlations between the weights are negligible.

The key idea here is that instead of viewing the weights of individuals as the input layer of a multi-layered classification network, we instead focus on the second-to-last layer. The move from that layer to the next final layer exactly encapsulates discrete choice based on linear utility and softmax, as in the Logit model. This leads to a couple of subtle implications.

1. In cases where the second-to-last layer is deep into the network, cross-weight correlations will be picked up along the network.
2. There is no guarantee that the distribution of the weights in the second-to-last layer produces a variance vector with high enough dimensionality (see the discussion in the previous Section 7.2.2), which is a necessary condition for the high-dimensional limit to hold properly. Utility, even when composed from a multitude of feature-weights, may end up having a low effective dimension of complexity.
3. The measurements become the edge weights of the neural network leading up to the final layer to each category (alternative). We may no longer be able to directly sample them, and the new measurements may be non-linear expressions of actual measurements.

In order to make sense of a preference market being generated directly for a utility function, we also require a concrete distribution on the alternatives, i.e., the neural network weights on the edges leading to the last layer. With that given, we are able to use PCA on the measurement space (modifying our new weights accordingly, further shifting their distribution), and finally, standardize the distributions as usual.

This leaves us with linear utility, but also with non-zero cross-weight correlations. As the non-linearity becomes ‘stronger’, one would expect the correlations to do so as well. Finally, we hypothesize that for linear models with low enough cross-weight correlations, one can bound the deviation from the correlation-free model we studied in this paper. This, however, is outside the scope of this paper and is left for future work to explore.

## Figures and Tables

**Table 1:** Summary of empirical preference data, sorted by comparison entropy.

Preference Market/Source of Data	η	Comparison Entropy	*A*	*I*
French approval ballots experiment	1.26	−0.046	16	409
Israeli city population	1.3	−0.058	100	9,662,037
Minneapolis mayoral elections	1.56	−0.150	35	80,101
Spotify top charts by country	0.65	−0.253	21	45
Jester joke set, high variance	2.22	−0.399	10	59,132
Israeli legislative elections	2.3	−0.427	13	4,690,442

**Figure A1:**
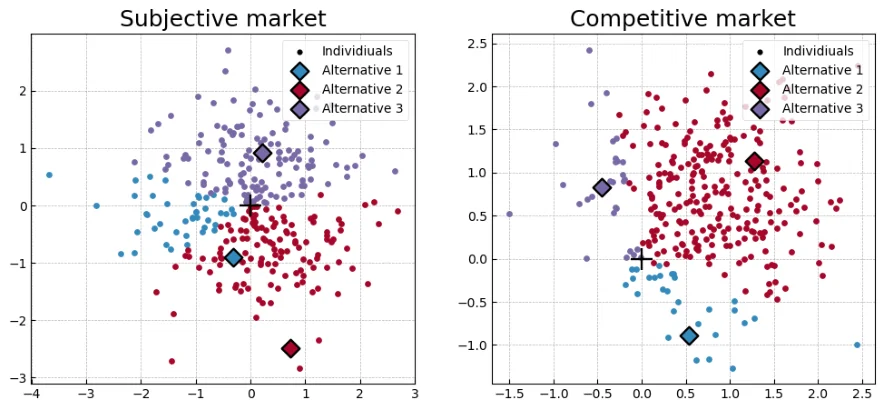
Segmentation of the weights-space $R^{F}$ by a market with three alternatives. Each alternative and its respective segment are colored accordingly. Small dots represent individuals’ weight vectors, colored by their preferred alternative. Diamonds represent the alternatives. The boundary of each segment must go through the center of axes, which is marked with a plus. For visual simplicity we choose F=2 (two features). Regions are created by a set of linear conditions ${\left\{{\bar{\omega }}_{i}\cdot {\bar{m}}_{a}>{\bar{\omega }}_{i}\cdot {\bar{m}}_{a^{\prime }}\right\}}_{a^{\prime }\neq a}$, which result in polyhedral cones in $R^{F}$ whose boundaries pass through the origin.

**Figure A2:**
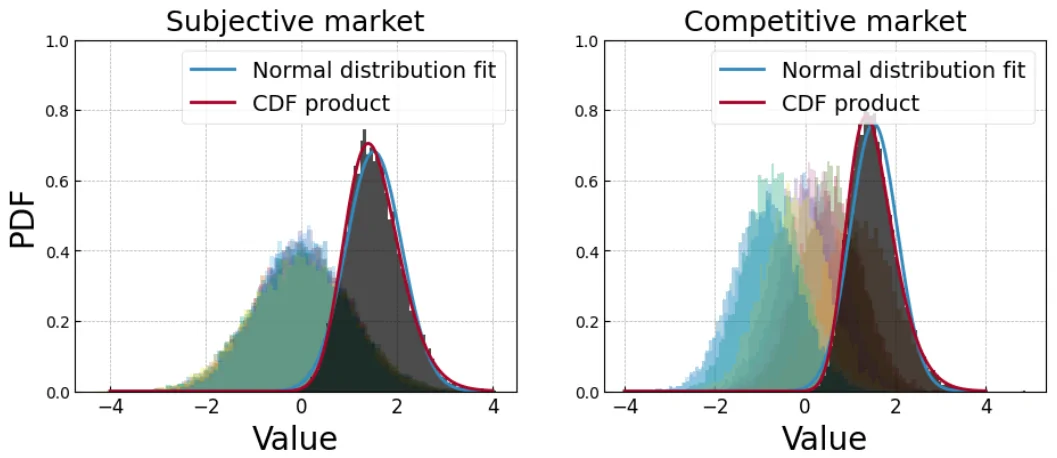
Comparing the distribution of assigned values $v_{i,a}$ (shaded in colors) and the distribution of market product (shaded black). Two methods of approximating the product are displayed: a normal distribution fit and a product of the CDFs of the alternatives’ values.

**Figure A3:**
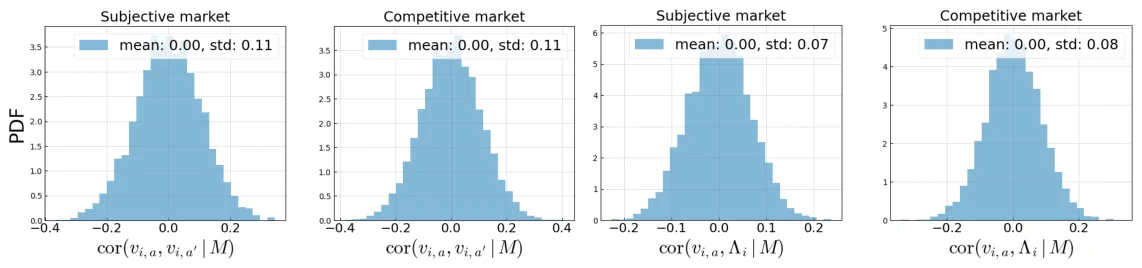
(**Left**): Distribution of $\mathrm{cor}(v_{i,a},v_{i,a^{\prime }}\;|\;M)$ with respect to M, with F=100. (**Right**): Distribution of $\mathrm{cor}(v_{i,a},\;\Lambda_{i}\;|\;M)$. As we approach the limiting behavior of the number of features being large, we do not necessarily see the correlations approaching zero, which implies a standard concentration of measure argument is not available. However, weakly correlated normal variables are roughly independent.

**Figure A4:**
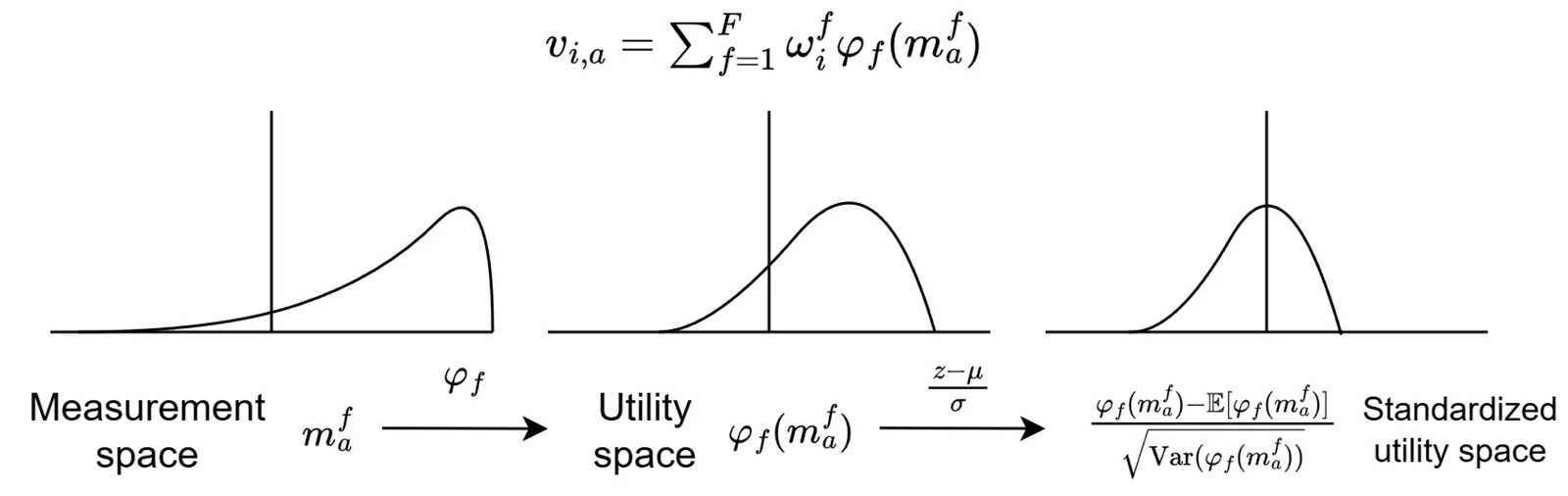
Turning real measurable data into standardized utilities.

## Data Availability

The data presented in this study are derived from resources publicly available in URLs provided in the body of the paper.

## Appendix A. Background: Definitions and Theorems

For completeness, we detail a few standard probability definitions and theorems [38].

**Definition** **A1** (Convergence in distribution)**.** *We say that a sequence ${\left\{X_{n}\right\}}_{n=1}^{\infty }$ of real-valued random variables with a cumulative distribution function (CDF) ${\left\{F_{n}\left(x\right)\right\}}_{n=1}^{\infty }$ converges in distribution (or that it has a limit in distribution) to a random variable X with CDF F(x) and denote $X_{n}\overset{d}{\to }X$ if*

(A1) $$\lim_{n\to \infty }F_{n}\left(x\right)=F\left(x\right)$$

*at every point x∈R where the function F is continuous.*

**Definition** **A2** (Convergence in probability)**.** *We say that a sequence ${\left\{X_{n}\right\}}_{n=1}^{\infty }$ of real-valued random variables converges in probability towards a random variable X, denoted $X_{n}\overset{p}{\to }X$, if for all ε>0 we have*

(A2) $$\lim_{n\to \infty }P(|X_{n}-X\left|>\varepsilon \right)=0.$$

*Convergence in probability is a stronger condition than convergence in distribution.*

**Theorem** **A1** (Lyapunov condition Lindeberg triangular array central limit theorem)**.** *Let $\left\{X_{n,k}\right\}$ be a triangular array, independent within each row, with $E[X_{n,k}]=0$ and $s_{n}^{2}=\sum_{k}\mathrm{var}X_{n,k}$. If for some δ>0,*

(A3) $$\frac{1}{s_{n}^{2+\delta }}\sum_{k=1}^{n}E[|X_{n,k}{|}^{2+\delta }]\underset{n\to \infty }{\to }0,$$

*then we have $\frac{1}{s_{n}}\sum_{k=1}^{n}X_{n,k}\overset{d}{\to }N\left(0,1\right)$.*

**Theorem** **A2** (Berry–Esseen non-uniform bound)**.** *Let $X_{1},X_{2},\ldots ,X_{n}$ be independent random variables with means $E[X_{i}]=0$, variances $\mathrm{Var}\left(X_{i}\right)=\sigma_{i}^{2}$, and third absolute moments $E[|X_{i}{|}^{3}]<\infty$. Define*

(A4) $$s_{n}^{2}=\sum_{i=1}^{n}\sigma_{i}^{2},\;Z_{n}=\frac{1}{s_{n}}\sum_{i=1}^{n}X_{i}.$$

*Let Φ(x) denote the standard normal cumulative distribution function. Then, there exists a constant C>0 such that for all x∈R,*

(A5)
$$\left|P\left(Z_{n}\leq x\right)-\Phi \left(x\right)\right|\leq \frac{C}{1+{\left|x\right|}^{3}}\frac{1}{s_{n}^{3}}\sum_{i=1}^{n}E[|X_{i}{|}^{3}].$$

**Lemma** **A1** (Multivariate normal probability density function)**.** *The probability density function for a multivariate normal variable $X\in R^{d},\;X∼N\left(\bar{\mu },\Sigma \right)$ is provided by*

(A6) $$\frac{\exp(-\frac{1}{2}{\left(\bar{x}-\bar{\mu }\right)}^{T}\Sigma^{-1}\left(\bar{x}-\bar{\mu }\right))}{\sqrt{{\left(2\pi \right)}^{d}\det\left(\Sigma \right)}}.$$

## Appendix B. Proof of Main Theorems

**Theorem** **A3.** *(Section 4.3 Equation* (18)*) Let $X=\left(X_{1},\ldots ,X_{K}\right)\in R^{K}$ be a random vector with independent elements $E[X_{k}]=0$ and $\mathrm{var}(X_{k})=1$. Let $Y=\left(Y_{1},\ldots ,Y_{K}\right)\in R^{K}$ be a random vector with independent elements, generated independently of X, with $E\left[Y_{k}\right]=\mu_{k}$ and $\mathrm{var}\left(Y_{k}\right)=\sigma_{k}^{2}$. Assume that ∥Y∥>0 almost surely, and define the normalized vector as*

$$\hat{Y}:=\frac{Y}{∥Y∥}\in S^{K-1}.$$

*Suppose that ${∥\mu ∥}^{2}+{∥\sigma ∥}^{2}=1$, $\sum_{k=1}^{K}E\left[Y_{k}^{4}\right]\to 0$, and*

$$\sum_{k=1}^{K}E[|{Y_{k}{|}^{3}]E[|X_{k}|}^{3}]\underset{K\to \infty }{⟶}0.$$

*Let $f_{K}:R^{K}\to R$ be a sequence of continuous functions. Then,*

(A7) $$\lim_{K\to \infty }\left|\int_{\Omega_{Y}}P_{X}\left(f_{K}\left(\hat{Y}\right)>X\cdot \hat{Y}\;|\;Y=y\right)dP\left(Y\right)-\int_{\Omega_{Y}}\Phi \left(f_{K}\left(\hat{Y}\right)\right)dP\left(Y\right)\right|=0,$$

*where* Φ *denotes the standard normal CDF.*

**Lemma** **A2.**
*Given the setting in Theorem A3, the following two conditions follow directly by applying Markov’s inequality:*

1. *$\sum_{k=1}^{K}E[|X_{k}{|}^{3}]|Y_{k}{|}^{3}\overset{p}{⟶}0$;*
2. *$\sum_{k=1}^{K}|X_{k}{|}^{3}E[|Y_{k}{|}^{3}]\overset{p}{⟶}0$.*

**Proof of Theorem** **A3.** First, since

$${E[∥Y∥}^{2}]=\sum_{k=1}^{K}\left(\mu_{k}^{2}+\sigma_{k}^{2}\right)=1,$$

and the elements of *Y* are independent, we have

$${\mathrm{var}(∥Y∥}^{2})=\sum_{k=1}^{K}\mathrm{var}\left(Y_{k}^{2}\right)\leq \sum_{k=1}^{K}E\left[Y_{k}^{4}\right]⟶0.$$

Therefore, ${∥Y∥}^{2}\overset{p}{\to }1$; hence, $∥Y∥\overset{p}{\to }1$. For every fixed realization Y=y, denote $\hat{y}=y/∥y∥$. The random variables $X_{1}{\hat{y}}_{1},\ldots ,X_{K}{\hat{y}}_{K}$ are independent, have mean zero, and their variances sum to $\sum_{k=1}^{K}{\hat{y}}_{k}^{2}=1$. Using the Berry–Esseen bound, there exists a universal constant *C* such that

(A8) $$\begin{array}{cc} \left|P_{X}\left(X\cdot \hat{y}<f_{K}\left(\hat{y}\right)\right)-\Phi \left(f_{K}\left(\hat{y}\right)\right)\right| & \leq C\frac{\sum_{k=1}^{K}E[|X_{k}{\hat{y}}_{k}{|}^{3}]}{1+|f_{K}\left(\hat{y}\right){|}^{3}}\\ \; & \leq \frac{C}{{∥y∥}^{3}}\sum_{k=1}^{K}|y_{k}{|}^{3}E[|X_{k}{|}^{3}]. \end{array}$$

By Lemma A2, the sum on the right converges to zero in probability, while ${∥Y∥}^{-3}\overset{p}{\to }1$. Therefore, the Berry–Esseen error converges to zero in probability. Since the error is bounded by 1, it also converges to zero in the mean. It follows that

$$\begin{array}{cc} \; & \left|\int_{\Omega_{Y}}P_{X}\left(X\cdot \hat{Y}<f_{K}\left(\hat{Y}\right)\;|\;Y=y\right)dP\left(Y\right)-\int_{\Omega_{Y}}\Phi \left(f_{K}\left(\hat{Y}\right)\right)dP\left(Y\right)\right|\\ \; & \;\leq \int_{\Omega_{Y}}\left|P_{X}\left(X\cdot \hat{y}<f_{K}\left(\hat{y}\right)\right)-\Phi \left(f_{K}\left(\hat{y}\right)\right)\right|dP\left(y\right)\underset{K\to \infty }{\to }0. \end{array}$$

□

**Corollary** **A1.**
*Under the same setup as in Theorem A3, given some Lipschitz function g:[0,1]→[0,1], we have*

(A9)
$$\lim_{K\to \infty }\left|\int_{\Omega_{Y}}g\left(P_{X}\left(X\cdot \hat{Y}<f_{K}\left(\hat{Y}\right)\;|\;Y=y\right)\right)dP\left(Y\right)-\int_{\Omega_{Y}}g\left(\Phi \left(f_{K}\left(\hat{Y}\right)\right)\right)dP\left(Y\right)\right|=0.$$

Indeed, if *L* is a Lipschitz constant of *g*, then

$$\left|g\left(P_{X}\left(X\cdot \hat{y}<f_{K}\left(\hat{y}\right)\right)\right)-g\left(\Phi (f_{K}\left(\hat{y}\right))\right)\right|\leq L\left|P_{X}\left(X\cdot \hat{y}<f_{K}\left(\hat{y}\right)\right)-\Phi \left(f_{K}\left(\hat{y}\right)\right)\right|,$$

and the result follows from the proof of Theorem A3.

**Theorem** **A4.** *(Section 4.3 Equation* (20)*) Given the assumptions on Y in Theorem A3, let $x\left(K\right)=\left(x_{1},\ldots ,x_{K}\right)$ be a deterministic sequence, and denote $s_{K}^{2}:=x^{2}\left(K\right)\cdot \sigma^{2}\left(K\right)$. Assume that, for some ε>0, $s_{K}^{2}>\varepsilon$ for all sufficiently large K, and that*

$$\sum_{k=1}^{K}|x_{k}{|}^{3}E[|Y_{k}{|}^{3}]\underset{K\to \infty }{⟶}0.$$

*Assume additionally that*

$$\underset{K}{\sup}\left|\frac{x(K)\cdot \mu (K)}{\sqrt{x^{2}\left(K\right)\cdot \sigma^{2}\left(K\right)}}\right|<\infty .$$

*Then,*

(A10)
$$\frac{x\left(K\right)\cdot \hat{Y}\left(K\right)-x\left(K\right)\cdot \mu \left(K\right)}{\sqrt{x^{2}\left(K\right)\cdot \sigma^{2}\left(K\right)}}\overset{d}{⟶}N\left(0,1\right)$$

*as K→∞. Here, $\mu \left(K\right)=\left(\mu_{1},\ldots ,\mu_{K}\right)$, $\sigma^{2}\left(K\right)=\left(\sigma_{1}^{2},\ldots ,\sigma_{K}^{2}\right)$, and $x^{2}\left(K\right)$ denotes the pointwise-squared vector of x(K).*

**Proof of Theorem** **A4.** For readability, we omit (K) from all *K*-length vectors, although they still depend on *K*. We first establish convergence for the pre-normalized vector *Y*, whose elements are independent. Let $s_{K}^{2}=x^{2}\cdot \sigma^{2}$. The random variables

$$x_{k}\left(Y_{k}-\mu_{k}\right),\;k=1,\ldots ,K$$

are independent, have mean zero, and their variances sum to $s_{K}^{2}$. Using ${\left|a-b\right|}^{3}\leq {4(|a|}^{3}+{\left|b\right|}^{3})$ and Jensen’s inequality, $|\mu_{k}{|}^{3}\leq E[|Y_{k}{|}^{3}]$, we obtain

$$E[|Y_{k}-\mu_{k}{|}^{3}]\leq 4\left(E[|Y_{k}{|}^{3}]+|\mu_{k}{|}^{3}\right)\leq 8E[|Y_{k}{|}^{3}].$$

We can then check for the Lyapunov condition,

$$\begin{array}{cc} \frac{1}{s_{K}^{3}}\sum_{k=1}^{K}E\left[|x_{k}\left(Y_{k}-\mu_{k}\right){|}^{3}\right] & =\frac{1}{s_{K}^{3}}\sum_{k=1}^{K}|x_{k}{|}^{3}E[|Y_{k}-\mu_{k}{|}^{3}]\\ \; & \leq \frac{8}{\varepsilon^{3/2}}\sum_{k=1}^{K}|x_{k}{|}^{3}E[|Y_{k}{|}^{3}]\underset{K\to \infty }{⟶}0. \end{array}$$

The Lyapunov condition thus holds; hence,

$$Z_{K}:=\frac{x\cdot Y-x\cdot \mu }{s_{K}}\overset{d}{⟶}N\left(0,1\right).$$

As shown in the proof of Theorem A3, $∥Y∥\overset{p}{⟶}1$. Since ∥Y∥>0 almost surely, it follows that ${∥Y∥}^{-1}\overset{p}{\to }1$. Using $\hat{Y}=Y/∥Y∥$, we may write

$$\begin{array}{cc} \frac{x\cdot \hat{Y}-x\cdot \mu }{s_{K}} & =\frac{1}{∥Y∥}\frac{x\cdot Y-x\cdot \mu }{s_{K}}+\frac{x\cdot \mu }{s_{K}}\left(\frac{1}{∥Y∥}-1\right)\\ \; & =\frac{1}{∥Y∥}Z_{K}+\frac{x\cdot \mu }{s_{K}}\left(\frac{1}{∥Y∥}-1\right). \end{array}$$

By Slutsky’s theorem,

$$\frac{1}{∥Y∥}Z_{K}\overset{d}{⟶}N\left(0,1\right).$$

Moreover, the sequence $|x\cdot \mu |/s_{K}$ is bounded by assumption, so

$$\frac{x\cdot \mu }{s_{K}}\left(\frac{1}{∥Y∥}-1\right)\overset{p}{⟶}0.$$

A second application of Slutsky’s theorem concludes the proof. □

## Appendix C. Market Segmentation

Consider a realized market, i.e., all measurements, provided by a matrix *M* were drawn. For each weight vector *w*, we note the alternative that maximized the value. This defines a function from the space of weight vectors, $R^{F}$, to {1…A}. The function separates (or segments) $R^{F}$ into disjoint regions according to its pre-image. In each region, all weight vectors share the same best alternative. The distribution of values within each region is not necessarily normal.

**Definition** **A3.**
*Segment. The segment of an alternative a in a realized market with measurements M is the set of all potential weight vectors that would prefer it over all others in the market,*

(A11)
$$S_{a}\left(M\right):=\left\{\bar{\omega }\in R^{F}:\bar{\omega }\cdot {\bar{m}}_{a}>\bar{\omega }\cdot {\bar{m}}_{a^{\prime }}\;\forall a^{\prime }\neq a\right\}.$$

*Note that the probability of a random weight vector being in the segment of alternative a is exactly its market share $T_{a}\left(M\right)$, and ${\bar{\omega }}_{i}\in S_{a}\left(M\right)\Leftrightarrow c_{i,a}=1$.*

**Definition** **A4.**
*Segmented values. The segmented value of an alternative in a realized market with measurements M is a random variable defined as the value an individual assigns to it, conditioned on the alternative being the individual’s best choice. More generally, we can define the value that an individual who picks alternative $a^{\prime }$ assigns to alternative aas*

(A12)
$$v_{i,a}^{*a^{\prime }}:={\bar{\omega }}_{i}\cdot {\bar{m}}_{a}\;\;\mathrm{for}\;{\bar{\omega }}_{i}\in S_{a^{\prime }}\left(M\right).$$

Conditioning the same way over $c_{i,a}=1$, we define the segmented weight means ${\bar{\mu }}_{W*a}$ and segmented weight variances ${\bar{\sigma }}_{W*a}^{2}$ as the vector means and variances of weight vectors in the segment of alternative *a*,

(A13) $$\mu_{W*a}^{f}=E_{{\bar{\omega }}_{i}}\left[\omega_{i}^{f}\;|\;M,c_{i,a}=1\right],\;{\left(\sigma_{W*a}^{f}\right)}^{2}={\mathrm{var}}_{{\bar{\omega }}_{i}}\left(\omega_{i}^{f}\;|\;M,c_{i,a}=1\right).$$

In any realized market with measurements *M*, we understand that

(A14) $${\bar{\mu }}_{W}=\sum_{a=1}^{A}T_{a}{\bar{\mu }}_{W*a},\;{\bar{\sigma }}_{W}^{2}=\sum_{a=1}^{A}T_{a}{\bar{\sigma }}_{W*a}^{2}+\sum_{a=1}^{A}T_{a}{\left({\bar{\mu }}_{W*a}-{\bar{\mu }}_{W}\right)}^{2}.$$

In other words, the sum of segmented means weighted by market share has to equal the population-wide mean for the weight vectors, and similarly for the variance. Let us now observe the value gained by any one individual from the market as a random variable.

**Definition** **A5.**
*Product and contribution. The contribution of alternative a is a random variable defined as $\lambda_{i,a}:=c_{i,a}{\bar{\omega }}_{i}\cdot {\bar{m}}_{a}$. The product of a market (market product as in the macro-economics definition of product, such as in the gross domestic product) is the sum of all the contributions, $\Lambda_{i}:=\sum_{a=1}^{A}c_{i,a}{\bar{\omega }}_{i}\cdot {\bar{m}}_{a}$, and it is equal to the value provided by the best alternative for a random individual. Note that both product and contributions are dependent on both ${\bar{\omega }}_{i}$ and M. We define the mean and variance of the product in a realized market with measurements M as follows:*

(A15)
$$\hat{\mu }\left(M\right):=E_{{\bar{\omega }}_{i}}\left[\Lambda_{i}\;|\;M\right]=E_{{\bar{\omega }}_{i}}\left[\sum_{a=1}^{A}c_{i,a}{\bar{\omega }}_{i}\cdot {\bar{m}}_{a}\;|\;M\right],\;{\hat{\sigma }}^{2}\left(M\right):={\mathrm{var}}_{{\bar{\omega }}_{i}}\left(\sum_{a=1}^{A}c_{i,a}{\bar{\omega }}_{i}\cdot {\bar{m}}_{a}\;|\;M\right).$$

The distribution of the market’s product is exactly a mixture distribution of the segmented value distributions from each alternative, or in other words, their contributions. We can express the mean and variance in terms of the segmented means and variances:

(A16) $$\hat{\mu }\left(M\right)=\sum_{a=1}^{A}T_{a}{\bar{m}}_{a}\cdot {\bar{\mu }}_{W*a},$$

(A17) $${\hat{\sigma }\left(M\right)}^{2}\approx \sum_{a=1}^{A}T_{a}\left({\bar{m}}_{a}^{2}\cdot {\bar{\sigma }}_{W*a}^{2}\right)+\left[\sum_{a=1}^{A}T_{a}{\left({\bar{m}}_{a}\cdot {\bar{\mu }}_{W*a}\right)}^{2}-\hat{\mu }{\left(M\right)}^{2}\right].$$

Note that the equation for the segmented variance is only an approximation, since conditioning weights on segments creates cross weight correlations.

In a random market,

(A18) $$E\left[\hat{\mu }\right]=\sum_{a=1}^{A}\int_{\Omega_{M}^{A}}\int_{\Omega_{W}}c_{i,a}\cdot {\bar{\omega }}_{i}\cdot {\bar{m}}_{a}\;dP\left({\bar{\omega }}_{i}\right)\;dP\left(M\right)=A\int_{-\infty }^{\infty }\Phi {\left(x\right)}^{A-1}\phi \left(x\right)x\;dx.$$

This value is exactly the expected value of the maximum between *A* independent standard normal variables. It grows roughly as $\sqrt{2\ln A}$ [39].

**Remark** **A1.**
*$\Lambda_{i}\left(M\right)$ is a mixture distribution of normal distributions.*

*Due to the segmentation of the market, $\Lambda_{i}\left(M\right)∼\sum_{a=1}^{A}T_{a}N({\bar{m}}_{a}\cdot {\bar{\mu }}_{W*a},\;{\bar{m}}_{a}^{2}\cdot {\bar{\sigma }}_{W*a}^{2})$, a mixture distribution of approximately normal distributions. (We do not prove the claim that the contributions being normal in this paper; it is nontrivial due to cross-weight correlations in the segments. Figure A2 in the next appendix shows the distributions of market product and of individual contributions, and in practice they appear as approximately normal.) While not a concise proof, it hints that the market product itself will end up having approximately a normal distribution (with respect to ${\bar{\omega }}_{i}$), as we will see in Appendix D.*

## Appendix D. Deriving the Market Share Distribution

For a realized market, each value $v_{i,a^{\prime }}={\bar{\omega }}_{i}\cdot {\bar{m}}_{a^{\prime }}$ is approximately normally distributed over $\Omega_{W}$, but not independent. If they were, however, we would be able to write a concise distribution for the maximal value for a random weight vector,

(A19) $$P\left(\max_{1\leq a\leq A}v_{i,a}<t\right)=\prod_{1\leq a\leq A}\Phi \left(\frac{t-{\bar{\mu }}_{W}\cdot {\bar{m}}_{a}}{\sqrt{{\bar{\sigma }}_{W}^{2}\cdot {\bar{m}}_{a}^{2}}}\right).$$

By comparing this expression with simulated data, we can see that it is fairly accurate even with dependence, as shown in Figure A2. This is justified by the fact that for a random market (which is just a randomly picked realized market),

(A20) $$E_{M}\left[{\mathrm{cov}}_{{\bar{\omega }}_{i}}\left(v_{i,a},v_{i,a^{\prime }}\;|\;M\right)\right]=E_{M}\left[{\bar{\sigma }}_{W}^{2}\cdot \left({\bar{m}}_{a}⊙{\bar{m}}_{a^{\prime }}\right)\right]=0.$$

Zero expected covariance does not by itself imply zero correlation in a realized market. However, $\mathrm{var}\left(v_{i,a}\;|\;M\right)={\bar{\sigma }}_{W}^{2}\cdot {\bar{m}}_{a}^{2}\overset{p}{\to }{\left\|{\bar{\sigma }}_{W}\right\|}^{2}>0$. It therefore remains to determine whether the covariance concentrates around zero as F→∞,

(A21) $${\mathrm{var}}_{M}\left({\mathrm{cov}}_{{\bar{\omega }}_{i}}\left(v_{i,a},v_{i,a^{\prime }}\;|\;M\right)\right)=E\left[{\left({\bar{\sigma }}_{W}^{2}\cdot \left({\bar{m}}_{a}⊙{\bar{m}}_{a^{\prime }}\right)\right)}^{2}\right]=\sum_{f=1}^{F}{\left(\sigma_{W}^{f}\right)}^{4},$$

where ⊙ denotes element-wise vector product. This is because any single factor of $m_{a}^{f}$ or $m_{a^{\prime }}^{f}$ in the expanded expression cancelsout the term it is a part of, and, furthermore, $E[{m_{a}^{f}}^{2}]=1$.

(A22) $$\frac{{\left\|{\bar{\sigma }}_{W}^{2}\right\|}^{2}}{{\left\|{\bar{\sigma }}_{W}\right\|}^{4}}=\frac{\sum_{f=1}^{F}{\left(\sigma_{W}^{f}\right)}^{4}}{{\left(\sum_{f=1}^{F}{\left(\sigma_{W}^{f}\right)}^{2}\right)}^{2}}.$$

If this ratio tends toward zero, Chebyshev’s inequality and Slutsky’s theorem imply that ${\mathrm{cor}}_{{\bar{\omega }}_{i}}\left(v_{i,a},v_{i,a^{\prime }}|M\right)\overset{p}{\to }0$. This ratio isthe inverse participation ratio (IPR) of the vector ${\bar{\sigma }}_{W}$ that measures the degree to which its components are spread around equally. The requirement for a vector product or weighted sum to tend to a normal distribution as the vector length increases, is that the variance is spread out equally and not clumped up at any one value. This should coincide with the idea that ${\bar{\sigma }}_{W}^{2}$ is not concentrated at any specific feature, but instead spread out evenly across them, which implies that multiplying every element by itself greatly reduces the resulting sum. As we see in Figure A3, this value does not necessarily vanish as F→∞, but it remains relatively small.

Recall Remark A1, which led us to suspect that the distribution of the market product $\Lambda_{i}$ will have a normal distribution. If that were the case, and we were to show that $\mathrm{cor}({\bar{\omega }}_{i}\cdot {\bar{m}}_{a},\;\Lambda_{i}\;|M)$ is small enough to treat these random variables as independent (which is what we observe in Figure A3), we would have

(A23) $$\begin{array}{cc} P(T_{a}<x) & \approx \int_{\Omega_{M}^{A}}P\left(\Phi \left(\frac{{\bar{\mu }}_{W}\cdot {\bar{m}}_{A+1}-{\hat{\mu }}_{A}}{\sqrt{{\bar{\sigma }}_{W}^{2}\cdot {\bar{m}}_{A+1}^{2}+{\hat{\sigma }}_{A}^{2}}}\right)<x\right)dP\left({\hat{\mu }}_{A},{\hat{\sigma }}_{A}^{2}\right)\\ \; & =\int_{\Omega_{M}^{A}}P\left(\frac{{\bar{\mu }}_{W}\cdot {\bar{m}}_{A+1}-{\hat{\mu }}_{A}}{\sqrt{{\bar{\sigma }}_{W}^{2}\cdot {\bar{m}}_{A+1}^{2}+{\hat{\sigma }}_{A}^{2}}}<\Phi^{-1}\left(x\right)\right)dP\left({\hat{\mu }}_{A},{\hat{\sigma }}_{A}^{2}\right), \end{array}$$

where ${\hat{\mu }}_{A}$ and ${\hat{\sigma }}_{A}^{2}$ are the (random variables) market product mean and variance of a random market of size *A*. Using a similar argument as in Theorem A4, as F→∞ we obtain

(A24) $$\begin{array}{cc} \; & \approx \int_{\Omega_{M}^{A}}\int_{-\infty }^{\infty }1\left\{\frac{z-{\hat{\mu }}_{A}}{\sqrt{{\left\|{\bar{\sigma }}_{W}\right\|}^{2}+{\hat{\sigma }}_{A}^{2}}}<\Phi^{-1}\left(x\right)\right\}\frac{\phi (\frac{z}{\left\|{\bar{\mu }}_{W}\right\|})}{\left\|{\bar{\mu }}_{W}\right\|}dz\;dP\left({\hat{\mu }}_{A},{\hat{\sigma }}_{A}^{2}\right),\\ \; & =\int_{0}^{\infty }\int_{-\infty }^{\infty }f_{{\hat{\mu }}_{A},{\hat{\sigma }}_{A}^{2}}\left({\hat{\mu }}_{A},{\hat{\sigma }}_{A}^{2}\right)\Phi \left(\frac{{\hat{\mu }}_{A}+\sqrt{{\left\|{\bar{\sigma }}_{W}\right\|}^{2}+{\hat{\sigma }}_{A}^{2}}\Phi^{-1}\left(x\right)}{\left\|{\bar{\mu }}_{W}\right\|}\right)d{\hat{\mu }}_{A}\;d{\hat{\sigma }}_{A}^{2}, \end{array}$$

where $f_{{\hat{\mu }}_{A},{\hat{\sigma }}_{A}^{2}}\left({\hat{\mu }}_{A},{\hat{\sigma }}_{A}^{2}\right)$ is the joint probability density function for the market product mean and variance (over $\Omega_{M}^{A}$). In theory, if each set of values ${\bar{\omega }}_{i}\cdot {\bar{m}}_{a}$ and ${\bar{\omega }}_{i}\cdot {\bar{m}}_{a^{\prime }}$ were to be fully independent, then naturally the maximum of disjoint subsets would also be independent. However, since we are working with non-zero correlations, there might be a compounding error. For that reason, we need to observe the correlation between a single value that an individual may get from some alternative, $v_{i,a}$ and the maximal value it may get from all the alternatives in some market, $\Lambda_{i}$, with respect to ${\bar{\omega }}_{i}$. Then, like in the previous case, we look at the expected value and the variance of this variable with respect to M. Note that we do this calculation with a market of size *A*, which does not include alternative *a*, making a total of A+1 alternatives.

(A25) $${\mathrm{cov}}_{{\bar{\omega }}_{i}}\left(v_{i,a},\Lambda_{i}\;|\;M\right)=\sum_{a^{\prime }\neq a}{\mathrm{cov}}_{{\bar{\omega }}_{i}}\left(v_{i,a},\lambda_{i,a^{\prime }}\;|\;M\right).$$

(A26) $${\mathrm{cov}}_{{\bar{\omega }}_{i}}\left(v_{i,a},\lambda_{i,a^{\prime }}\;|\;M\right)=E_{{\bar{\omega }}_{i}}\left[\left({\bar{\omega }}_{i}\cdot {\bar{m}}_{a}\right)\lambda_{i,a^{\prime }}\;|\;M\right]-E_{{\bar{\omega }}_{i}}\left[{\bar{\omega }}_{i}\cdot {\bar{m}}_{a}\;|\;M\right]E_{{\bar{\omega }}_{i}}\left[\lambda_{i,a^{\prime }}\;|\;M\right].$$

It is simple to show that the expected value of this expression over M is zero. However, calculating the correlation is more difficult, and so is showing that the variance of the correlation is small enough for us to treat ${\bar{\omega }}_{i}\cdot {\bar{m}}_{a}$ and $\Lambda_{i}$ as independent random variables. We will not be deriving a proof of this correlation being small in this paper, instead we only demonstrate it in simulations, as seen in Figure A3.

## Appendix E. Measurement Space and Utility Space

In this appendix, we discuss the concept of a ’utility space’, wherein we can conceptually measure utility as a linear quantity. Assume there are some bijective maps $\varphi_{f}:M_{f}\to U_{f}\subseteq R$ that translate each measurement into utility space $U=\prod_{f=1}^{F}U_{f}\subseteq R^{F}$. Since the map is bijective, it is invertible, which implies monotonicity when continuous. Without loss of generality we take it to be monotonically increasing. The distribution of measurements then becomes $P\left(\varphi_{f}\left(m_{a}^{f}\right)<x\right)=P\left(m_{a}^{f}<\varphi_{f}^{-1}\left(x\right)\right)$. This leaves us with a set of measurements that contribute to the total value in a linear manner, which relaxes the required assumption on linearity and independence to only having to assume that measurements are independent. For the sake of simplicity, we keep referring to measurements as $m_{a}^{f}$, even in utility space, as there is no particular reason to use the measurements before their projection into utility space.

When working with real data, since the $\varphi_{f}$ are unknown, we might consider using a discrete measurement space, which is much easier to explore bijections on. For example, if $M_{f}=\left\{0,1\right\}$ and P(x=1)=p, we have

$$\varphi_{2,p}\left(x\right)=\left\{\begin{array}{cc} \sqrt{\frac{1-p}{p}} & x=1\\ -\sqrt{\frac{p}{1-p}} & x=0. \end{array}\right.$$

This function is well defined for each 0<p<1 and is unique under the assumptions that it is (monotonically) increasing $E[\varphi_{2,p}\left(x\right)]=0$ and $\mathrm{var}(\varphi_{2,p}\left(x\right))=1$.
